# Foxj1a is expressed in ependymal precursors, controls central canal position and is activated in new ependymal cells during regeneration in zebrafish

**DOI:** 10.1098/rsob.170139

**Published:** 2017-11-22

**Authors:** Ana Ribeiro, Joana F. Monteiro, Ana C. Certal, Ana M. Cristovão, Leonor Saúde

**Affiliations:** 1 Instituto de Medicina Molecular, Faculdade de Medicina da Universidade de Lisboa, 1649-028 Lisboa, Portugal; 2 Instituto de Medicina Molecular e Instituto de Histologia e Biologia do Desenvolvimento, Faculdade de Medicina da Universidade de Lisboa, 1649-028 Lisboa, Portugal; 3 Champalimaud Research, Champalimaud Centre for the Unknown, Lisboa, Portugal

**Keywords:** Foxj1a, Shh, ependymal radial glia, CSF-contacting neurons, spinal cord injury, zebrafish

## Abstract

Zebrafish are able to regenerate the spinal cord and recover motor and sensory functions upon severe injury, through the activation of cells located at the ependymal canal. Here, we show that cells surrounding the ependymal canal in the adult zebrafish spinal cord express Foxj1a. We demonstrate that ependymal cells express Foxj1a from their birth in the embryonic neural tube and that Foxj1a activity is required for the final positioning of the ependymal canal. We also show that in response to spinal cord injury, Foxj1a ependymal cells actively proliferate and contribute to the restoration of the spinal cord structure. Finally, this study reveals that Foxj1a expression in the injured spinal cord is regulated by regulatory elements activated during regeneration. These data establish Foxj1a as a pan-ependymal marker in development, homeostasis and regeneration and may help identify the signals that enable this progenitor population to replace lost cells after spinal cord injury.

## 1. Introduction

The spinal cord develops from the embryonic neural tube, an initially homogeneous neuroepithelium. During development, progenitor cells in the neural tube acquire different identities depending on their position along the dorsoventral axis. The roof plate on the dorsal side (via BMPs and Wnts) and the floor plate on the ventral side (via Shh) provide positional information that assigns cells to distinct progenitor domains [1,2]. Each progenitor domain can give rise to several cell fates that emerge sequentially. The initial step in neuroepithelial patterning specifies different neuronal subtypes. Soon after, the progenitor region switches from a neuronal to a glial fate and begins generating oligodendrocytes and astrocytes that support neurons [3]. After the differentiation and migration of neurons and glial cells from the neuroepithelium, the remaining cells now lining the central canal give rise to an additional cell fate—ependymal cells. Ependymal cells (i.e. all the cells that contact the central canal) descend predominantly from the ventral progenitor domains p2 and pMN [4,5] and the floor plate [6], and require the ventral signal Shh for their correct specification [7].

As the remnant of the embryonic neuroepithelium, the ependymal region in the adult spinal cord retains not only the pseudo-stratified epithelial organization, but also a stem/progenitor character. These cells have neurosphere-forming capacity and are able to give rise to neurons, astrocytes and oligodendrocytes *in vitro*, both in mouse [8,9] and human [10,11]. By contrast, *in vivo* ependymal cells display a more restricted lineage potential, in the context of spinal cord injury. Upon damage to the spinal cord ependymal cells show a strong proliferative response, but fail to generate neurons, forming mostly astrocytes that incorporate the glial scar and a small number of oligodendrocytes [9,12]. The discrepancy between the *in vitro* and *in vivo* data suggests that neural stem cells in the ependymal region have the potential to replace all lost cells, including neurons, but the neuronal fate is inhibited by the microenvironment in the injured spinal cord. Therefore, if these inhibitory signals were removed it could be possible to direct the endogenous stem cells towards a neuronal lineage.

In contrast to mammals, adult zebrafish are able to efficiently regenerate the spinal cord due to its ability to regrow damaged axons and form new neurons, while avoiding the formation of a glial scar [13]. Injury-induced neurons arise from the ependymal region [14], suggesting that stem/progenitor cells in the zebrafish spinal cord have a wider regenerative potential than in mammals and could be used to identify the signals that help promote the neuronal fate.

The study of the behaviour of zebrafish ependymal cells during regeneration would be facilitated by the use of ependymal-specific molecular markers, but these are limited in zebrafish. A good candidate is Foxj1, which is specifically expressed by ependymal cells in the mouse spinal cord [9] and is also detected in the ependymal zone in the human spinal cord [15]. Moreover, Foxj1-expressing cells were shown to enter into a proliferative state after spinal cord injury, contributing mainly to astrocytes *in vivo* but with the ability to differentiate into other cell types, including neurons, when cultured *in vitro* [9]. In zebrafish, the Foxj1 homologue—Foxj1a—is expressed in the floor plate of the developing spinal cord [16] and was shown to be elevated after injury in embryos [17]. Yet, the cellular details of the Foxj1a distribution and response to injury in the adult spinal cord were not explored.

In this study we determined if Foxj1a can be used to identify ependymal cells and whether Foxj1a-expressing cells participate in the repair of the spinal cord. We report that Foxj1a expression in the ependymal region is conserved in zebrafish and accompanies ependymal cells from their genesis until adulthood. We also show that Foxj1 activity is important for the formation of the central canal, through the modulation of the Shh signalling pathway. Moreover, we confirm that Foxj1a-positive cells expand in response to injury through a Shh-dependent mechanism and contribute to the restoration of the spinal cord structure in zebrafish.

## 2. Results

### 2.1. Adult zebrafish ERGs express Foxj1a

**Figure 1.**
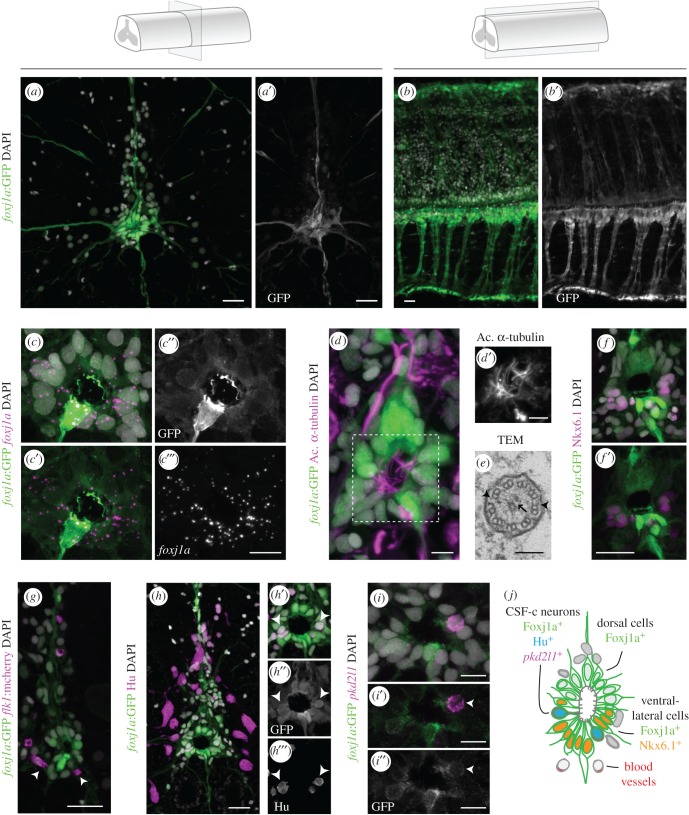
Foxj1a is expressed in ependymal cells in the zebrafish adult spinal cord. (*a*–*b*′) Confocal stack projection of a spinal cord transverse section (*a*,*a*′) and sagittal section (*b*,*b*′) in adult *Tg(0.6foxj1a:GFP)* transgenic zebrafish. (*c*–*c*‴) FISH of *foxj1a* (magenta) in a transverse section of a spinal cord expressing the *foxj1a*:GFP reporter (green), showing a similar pattern of expression. (*d*,*d*′) Immunostaining with acetylated α-tubulin (magenta) to label cilia present on the apical surface of Foxj1a-expressing cells (green). (*e*) TEM image of a cilium at the apical ependymal region, with a central pair of microtubules (arrow) and outer dynein arms (arrowheads). (*f*,*f*′) Ventral *foxj1a*:GFP^+^ cells express the progenitor marker Nkx6.1 (magenta). (*g*) BVs (mCherry^+^ endothelial cells in magenta) are present in the proximity of GFP^+^ ERGs (arrowheads). (*h*) Pan-neuronal marker HuC/D (Hu, magenta) immunostaining and *foxj1a*:GFP expression in a spinal cord transverse section. (*h*′–*h‴*) Magnification of the central canal highlighting Hu/Foxj1a double positive cells (arrowheads). (*i*–*i*″) FISH of *pkd2l1* (magenta) showing co-expression with *foxj1a*:GFP (arrowheads) in the ependymal region. (*j*) Scheme of the spinal cord ependymal region showing subtypes of Foxj1a-expressing cells, based on molecular expression and cell morphology. The term ependymal cells is not consensual, as other authors suggest that adult zebrafish spinal cords only contain ERGs. DAPI-labelled nuclei are shown in grey. Scale bars: 20 µm in *a*,*b*,*f–h*; 10 µm in *c*‴; 5 µm in *d*; 100 nm in *e*; 10 µm in *i*.

To determine if Foxj1a is expressed in the adult zebrafish spinal cord, we used the reporter transgenic line *Tg(0.6foxj1a:GFP)*, in which a small enhancer sequence drives the expression of the fluorescent protein GFP [18]. This transgenic line was shown to reproduce Foxj1a endogenous expression in several tissues, including the developing neural tube. In transverse sections of the adult spinal cord, the Foxj1a reporter labelled most cells surrounding the central canal—ependymal cells (figure 1*a*,*a*′). The majority of GFP^+^ ependymal cells had ependymo-radial glia (ERG) morphology [19], with projections that extended to the pial surface (figure 1*a*–*b*′). We also confirmed by fluorescent *in situ* RNA hybridization (FISH) on transgenic sections that the distribution of the *foxj1a*:GFP reporter is similar to the endogenous *foxj1a* gene, which is also detected in the cells surrounding the central canal (figure 1*c*–*c*‴).

A general feature of ependymal cells is the presence of cilia, and Foxj1(a) is associated with the formation of motile cilia [16,20–22]. To determine if Foxj1a^+^ ependymal cells in zebrafish are ciliated, we labelled cilia with acetylated α-tubulin, which revealed long cilia projecting into the central canal (figure 1*d*,*d*′). At a closer view, we detected one or two cilia per ependymal cell (electronic supplementary material, figure S1). Transmission electron microscopy (TEM) revealed that most cilia at the apical edge of cells around the central canal had a central pair of microtubules (*n* = 21/25) (figure 1*e*, arrow), as well as outer dynein arms (figure 1*e*, arrowhead). These results confirm that cells lining the central canal have long and likely motile cilia, features that are consistent with the expression of Foxj1a.

The adult zebrafish ependymal region was previously shown to retain an embryonic-like pattern of expression of several progenitor markers [23]. To assess the dorsoventral identity of Foxj1a^+^ cells, we tested the expression of ventral marker Nkx6.1. This analysis showed the expression of Foxj1a in both the Nkx6.1^+^ ventral half of the ERGs and in the Nkx6.1-negative dorsal region that corresponded to a Pax6^+^ domain (figure 1*f*,*f*′). Foxj1a expression was also more widespread in the ependymal region than that of other molecular markers associated with radial glial cells (GFAP and vimentin; electronic supplementary material, figure S2). Foxj1a^+^ ERGs were also in close proximity to blood vessels (BVs) (figure 1*g*; electronic supplementary material, figure S2), which provide support to neural stem cell niches [24,25]. These data settle Foxj1a as a general ependymal marker that labels a heterogeneous group of immature/progenitor cells.

Although the majority of cells surrounding the central canal had a radial glial morphology, a subset of *foxj1a*:GFP-expressing cells displayed a more rounded morphology with a bulbous apical attachment (figure 1*h*″). This morphology is reminiscent of cerebrospinal-fluid-contacting neurons (CSF-cNs) [26]. To test if Foxj1a is expressed in CSF-cNs, we examined the expression of the early neuronal marker HuC/D and a CSF-cN-specific marker, polycystic kidney disease 2-like 1 (PKD2L1) [26]. This analysis showed that *foxj1a*:GFP^+^ cells with CSF-cN morphology express HuC/D (figure 1*h*–*h*‴) and *pkd2l1* (figure 1*i*–*i*″), indicating that Foxj1a is expressed not only in ERGs, but also in CSF-cNs.

This initial characterization of Foxj1a expression in the adult zebrafish spinal cord demonstrated that, as in mammals, Foxj1a is expressed in the cells that line the central canal, which we broadly term as ependymal cells. The Foxj1a^+^ population is composed of various cell types, including different subtypes of radial glial progenitors and more differentiated CSF-cNs (figure 1*j*). However, despite the common expression of Foxj1, zebrafish and mammalian ependymal cells show some differences, namely in the distribution of markers such as GFAP (absent in mouse) and vimentin (more widespread in mouse) and in the proportion of cells with radial morphology (lower numbers in mouse) [9].

### 2.2. Foxj1a is expressed in the ERG precursors in the developing neural tube

We next asked if Foxj1a plays a role in the assembly of the ependymal region during neural tube development. Foxj1(a) is expressed in the embryonic floor plate [16,27] and roof plate [28]. In zebrafish, floor plate and roof plate cells participate in contraction of the primitive lumen to form the central canal [29], which occurs between 48 and 72 hours post-fertilization (hpf) as neural precursors exit the ventricular zone [30].

**Figure 2.**
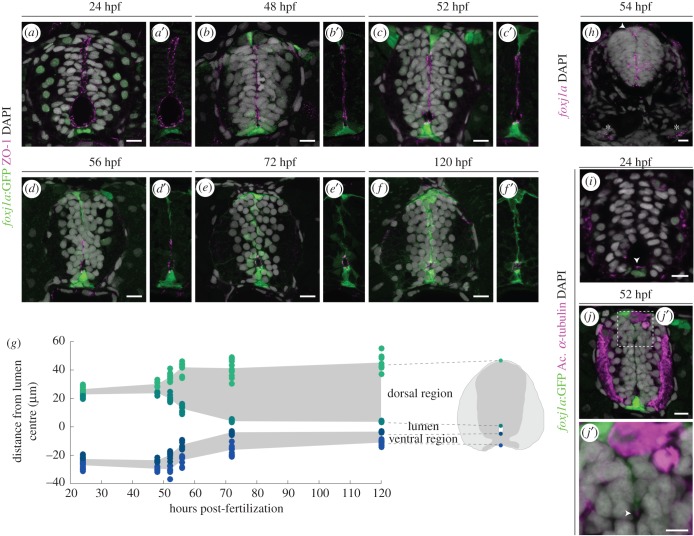
Foxj1a^+^ cells participate in the formation of the spinal cord central canal. (*a–f*′) Representative images of the neural tube region in transverse sections of *Tg(0.6foxj1a:GFP)* transgenic zebrafish embryos/larvae ranging from 24 to 120 hpf. The apical edge of the cells surrounding the lumen is identified by ZO-1 immunostaining (magenta) and the GFP reporter labels Foxj1a-expressing cells (green). (*g*) Quantification of lumen closure from 24 to 120 hpf. The positions of the floor plate, ventral and dorsal points of the lumen and roof plate are normalized to the middle point of the lumen. Sample number: 24 hpf (*n* = 12); 48 hpf (*n* = 6); 52 hpf (*n* = 9); 56 hpf (*n* = 9); 72 hpf (*n* = 11); 120 hpf (*n* = 8). (*h*) Confocal image of a FISH of *foxj1a* (magenta) in a 54 hpf embryo. *foxj1a* is expressed in the floor plate, ventro-lateral cells, roof plate (arrowhead) and pronephric (asterisks). (*i*,*j*,*j*′) Long cilia (arrowhead), labelled by acetylated α-tubulin (magenta), are present in Foxj1a-positive floor plate cells at 24 hpf (*i*) and in Foxj1a-expressing roof plate cells in the roof plate at 52 hpf (*j*,*j*′). DAPI-labelled nuclei are shown in grey. Scale bars: 10 µm in *a–j*; 5 µm in *j*′.

To determine if Foxj1a is expressed in the cells that help form the central canal, we examined the behaviour of *foxj1a*:GFP^+^ cells during this embryonic period. The tight-junction protein ZO-1 was used to reveal the outline of the primitive lumen. Expression of the reporter transgene was detectable in the floor plate at 24 hpf (figure 2*a*,*a′*) and continued to be expressed in the ventral midline at the subsequent time-points analysed (figure 2*b–f′*). At 48 hpf *foxj1a*:GFP started to be expressed in roof plate cells (figure 2*b*,*b′*), indicating that the expression pattern observed in chick is conserved in zebrafish [28]. Endogenous *foxj1a* expression in the roof plate was confirmed by FISH in sections of 54 hpf embryos (figure 2*h*). However, the analysis of the distribution of *foxj1a* transcripts also uncovered a domain of *foxj1a* expression that was not reproduced by the reporter transgene—a region of strong expression in the middle of the neural tube (figure 2*h*; electronic supplementary material, figure S3). Nevertheless, the reporter could still be used to follow the behaviour of the roof plate and floor plate during lumen contraction. At 52 hpf, the roof plate started to extend as the primitive lumen started to contract, and the apical projection of roof plate cells continued to stretch until the conversion of the lumen into the central canal was completed by 72 hpf (figure 2*c*–*e′*). By contrast, the floor plate showed little change during this process. The quantification of the size of the lumen in different embryos showed that the reduction of the lumen reproducibly occurred from 48 to 72 hpf, with a dorsal-to-ventral zipping mechanism (figure 2*g*). After lumen contraction at 72 hpf, the size of the ventral and dorsal regions remained constant until the last time-point analysed, 120 hpf (figure 2*f*,*f′*).

The initiation of Foxj1a expression in the neural tube also coincided with the appearance of long cilia. When Foxj1a started to be expressed in the floor plate (24 hpf) and the roof plate (48 hpf), GFP^+^ cells exhibited long cilia (figure 2*i–j′*). The correlation between Foxj1a expression and appearance of long cilia is consistent with the role of Foxj1a in the regulation of motile ciliogenesis.

These results show that Foxj1a is expressed in ERG precursors in the floor plate, roof plate and in cells close to the lumen in the middle region, which continue to surround the central canal after lumen contraction. These data also revealed that the *foxj1a*:GFP reporter transgene is expressed only in a subset of the endogenous *foxj1a* domains (floor plate and roof plate) in the neural tube, indicating that the enhancer in the construct lacks some of the *foxj1a* regulatory elements.

### 2.3. Loss of Foxj1a activity affects the timing of lumen contraction

**Figure 3.**
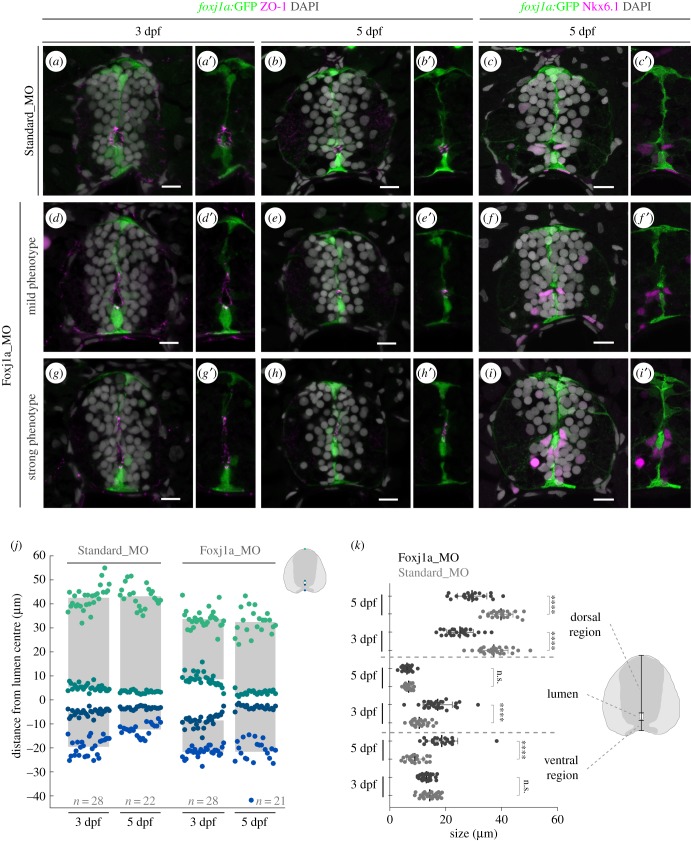
Neural tube lumen closure is perturbed in the absence of Foxj1a. (*a–i*′) Representative images of transverse sections of the neural tube of *Tg(0.6foxj1a:GFP)* transgenic larvae injected with Standard Morpholino (MO) (*a–c*′) or *foxj1a* MO (*d–i*′) at one-cell stage and analysed at 3 and 5 dpf. *foxj1a* MO-injected larvae showed lumen closure phenotypes with variable degrees of severity, between mild (*d–f*′) and strong (*g–i*′), as highlighted by the apical ZO-1 immunostaining (magenta) (*a*,*b*′,*d*,*e*′,*g*,*h*′). (*c*,*c*′,*f*,*f*′,*i*,*i*′) Nkx6.1^+^ cells (magenta) are still present lining the ventral half of the central canal in *foxj1a* morphants. DAPI-labelled nuclei are shown in grey. Scale bars: 10 µm. (*j*) Quantification of lumen closure in Standard MO and *foxj1a* MO larvae at 3 and 5 dpf. The positions are normalized to the middle point of the lumen. Sample number is shown in the graph and includes data from three independent experiments. (*k*) Quantification of the size of the lumen and the ventral and dorsal regions, derived from the data shown in (*j*). Each point represents one individual and the mean and s.d. bars are also shown. *p* values calculated using two-tailed unpaired *t*-test (****p < 0.0001; n.s., not significant).

To test if Foxj1a is required for the transition from primitive lumen to central canal, we examined the morphology of the lumen (visualized with ZO-1) in larvae injected with antisense morpholino oligonucleotides (MO) designed to block Foxj1a protein translation by targeting the start codon [16,31,32] (electronic supplementary material, figure S3). We first analysed larvae at 3 days post-fertilization (dpf), which showed a round and narrow lumen when injected with a standard control MO (figure 3*a*,*a′*). By contrast, *foxj1a* MO-injected larvae exhibited an elongated lumen, indicating that lumen contraction was delayed when compared to the controls (figure 3*d*,*d′*,*g*,*g′*). The degree of the delay varied between morphant larvae, but the size of the lumen was significantly larger and the dorsal region significantly smaller when compared to controls (figure 3*j*,*k*). These data suggest that the delay in lumen contraction observed in morphants results from a lagging dorsal zipping.

To determine if *foxj1a* morphants are able to complete the zipping of the lumen, we analysed MO-injected larvae at 5 dpf. At this stage control larvae displayed a small and ventrally positioned central canal (figure 3*b*,*b′*). In morphant larvae, the size of the lumen was equivalent to controls, but the position of the canal was altered (figure 3*j*,*k*). In *foxj1a* morphants with a milder phenotype, the position of the central canal was only modestly dorsalized (figure 3*e*,*e′*), but in more severe phenotypes the canal was displaced to the middle of the spinal cord (figure 3*h*,*h′*). The measurement of the size of the ventral region showed that floor plate cells in the morphant had stretched significantly (figure 3*j*,*k*). These results indicate that the lumen size phenotype observed in 3 dpf morphants is caused by a delay that ultimately affects the final position of the canal. The co-injection of the *foxj1a* MO with a MO-resistant form of the *foxj1a* mRNA resulted in the rescue of the curved body phenotype and the position of the central canal in a subset of larvae (electronic supplementary material, figures S4 and S5). Moreover, the injection of Cas9/gRNA complexes targeting the *foxj1a* locus resulted in a small number of larvae with a curved body and a central canal shifted dorsally (electronic supplementary material, figure S6). Together, these data argue that the morphant phenotype is specific to Foxj1a downregulation.

To determine if the dorsalization of the central canal in *foxj1a* morphants affects the identity of the cells encircling the canal, we analysed the expression of the ventral marker Nkx6.1. At 5 dpf, Nkx6.1 was expressed in cells lining the ventral side of the central canal in both control and morphant larvae (figure 3*c*,*c′*,*f*,*f′*,*i*,*i′*), suggesting that the dorsoventral identity of ependymal cells was not changed by the loss of Foxj1a function.

Together, these results indicate that Foxj1a activity influences the position of the central canal, but not the specification of the ependymal precursor cells surrounding the canal.

### 2.4. Foxj1a attenuates Shh activity and prolongs the proliferative phase

**Figure 4.**
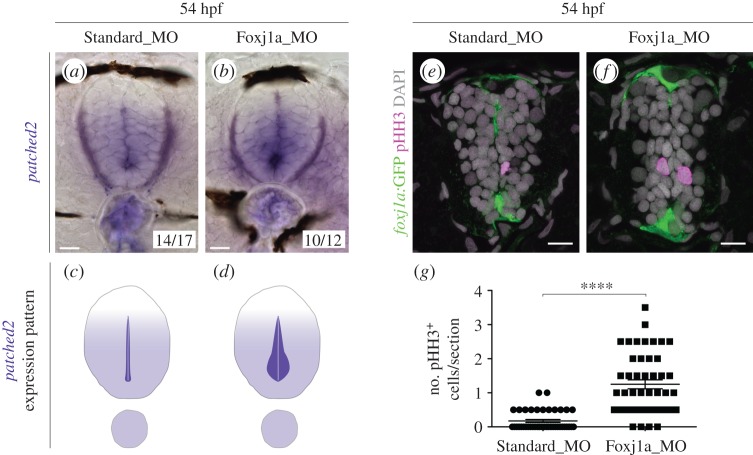
Shh signalling and proliferation are enhanced in the absence of Foxj1a. (*a*,*b*) Expression of *patched2* by ISH in 54 hpf embryos injected with Standard Morpholino (MO) (*a*) or *foxj1a* MO (*b*). Two types of expression patterns were observed, which are schematically represented in (*c*,*d*). The majority of *foxj1a* morphants display increased *patched2* expression in ventral cells surrounding the lumen. (*e*,*f*) Confocal images of transverse sections of *Tg(0.6foxj1a:GFP)* 54 hpf embryos injected with Standard MO (*e*) or *foxj1a* MO (*f*) and immunostained against phospho-histone H3 (pHH3) to detect mitotic cells (magenta). The *foxj1a*:GFP reporter is shown in green and DAPI-labelled nuclei are shown in grey. Scale bars, 10 µm. (*g*) Quantification of the number of pHH3^+^ cells per section (Standard MO, *n* = 43; *foxj1a* MO, *n* = 46). Data obtained from three independent experiments. Each point represents the average of two non-consecutive sections per embryo and the mean and s.d. bars are also shown. *p* value calculated using the two-tailed unpaired *t*-test (****p < 0.0001).

The lumen closure phenotype observed in *foxj1a* morphants was reminiscent of larvae lacking the microRNA *miR-219* [30]. In *miR-219* morphants, the extended lumen was associated with enhanced Shh activity and Shh-induced proliferation [33]. To test if Foxj1a also affects the activity of the Shh pathway, we assayed the expression of the Shh target gene *patched2* [34] by FISH in 54 hpf embryos, when the lumen is closing. Standard MO-injected larvae presented a ventral-to-dorsal accumulation of *patched2* transcripts near the lumen (figure 4*a*,*c*), which was increased in *foxj1a* morphants (figure 4*b*,*d*). This increase in the expression of a Shh target gene in the absence of Foxj1a is consistent with the hypothesis that Foxj1 dampens Shh signal transduction [27]. The increase in *patched2* expression is not a result of increased *shh* levels in the floor plate of *foxj1a* morphants, as assessed by FISH (electronic supplementary material, figure S7).

The Shh-signalling pathway has both patterning and mitogenic roles in the neural tube [35] and the attenuation of Shh activity is required for progenitor cells to exit the cell cycle and differentiate [33]. To test if the loss of Foxj1a affects the transition from proliferation to differentiation, we quantified the number of mitotic cells (labelled by phospho-histone H3(pHH3)) in control and morphant 54 hpf embryos. At this stage, very few mitotic cells were detected in the neural tube of Standard MO embryos (0.1744 ± 0.0437 cells/section) (figure 4*e*,*g*), but in *foxj1a* morphants the number of pHH3^+^ cells was significantly increased (1.25 ± 0.1326 cells/section) (figure 4*f*,*g*). This result suggests that a decrease in Foxj1a activity leads to an extended period of neural progenitor proliferation, likely due to the ectopic activation of the Shh pathway. This delay in the transition to differentiation may explain the delay in lumen closure observed in Foxj1a morphants. Together, these data propose a role for Foxj1a in the triggering of progenitor cell cycle exit and induction of lumen contraction, through the modulation of the Shh signal transduction.

### 2.5. Foxj1a-expressing cells participate in the regeneration of the spinal cord

**Figure 5.**
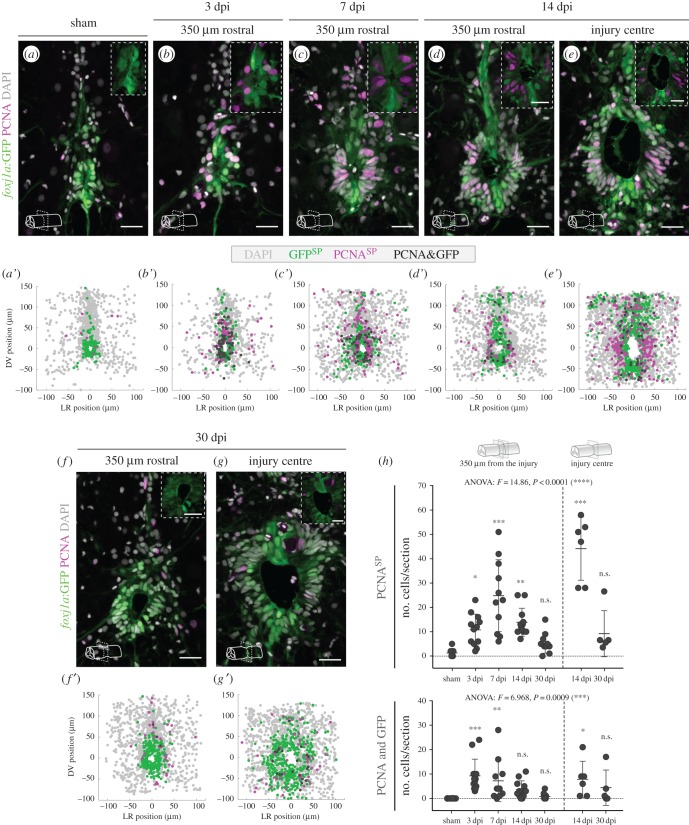
Spinal cord injury in adult zebrafish triggers proliferation of Foxj1a^+^ ERGs. (*a–g*) Representative confocal images of transverse sections of spinal cords of *Tg(0.6foxj1a:GFP)* transgenic zebrafish from 3 to 30 dpi. The control spinal cord in (*a*) was collected 7 days after a sham injury. The sections were located at 350 µm rostral to the injury centre (*b–d*,*f*) or at the injury site (*e*,*g*). Proliferative cells (in magenta) were labelled using an antibody against PCNA and cells expressing the GFP reporter are shown in green. DAPI-labelled nuclei are shown in grey. Scale bars, 20 µm. (*a*′–*g*′) Composite maps of cell positions quantified in transverse section images of different spinal cords for each time point (sham, 7 dpi, 14 dpi: *n* = 6; 3 dpi, 30 dpi: *n* = 5). DV, dorsal/ventral; LR, left/right; SP, single positive. (*h*) Quantification of the number of PCNA^SP^ (top plot) and PCNA^+^/GFP^+^ (bottom plot) cells per section. Each circle represents one section at 350 µm rostral or caudal from the injury centre (left side of plot) or at the injury site (right side of plot). The mean and s.d. bars are also shown and a one-way ANOVA *F*-test was performed between groups, followed by a Dunnett test to compare every mean to the control mean (*p < 0.05; **p < 0.01; ***p < 0.001; n.s., non-significant).

To determine if adult zebrafish Foxj1a^+^ ependymal cells contribute to the repair of the spinal cord after an injury, we examined the proliferative response of Fox1a^+^ cells after a compression injury protocol. In this protocol, the vertebral column was exposed and the spinal cord was compressed along the dorsoventral axis using forceps [36]. The site of the injury and the adjacent rostral and caudal regions (350 µm from the injury centre) were analysed in transverse sections. Proliferation was assayed using proliferating cell nuclear antigen (PCNA) antibody to label cells in G1 and S phases of the cell cycle [14]. In adult fish that had undergone only sham-injury, the number of PCNA^+^ cells was very low (figure 5*a*). The quantification of number and position of PCNA and GFP-expressing cells, plotted in a composite map of all samples, showed *foxj1a*:GFP^+^ cells around the central canal and very few PCNA^+^ cells (figure 5*a′*,*h*). At 3 and 7 days post-injury (dpi), the sections at the injury site were too disrupted to analyse; therefore, only the adjacent sections were quantified. At 3 dpi the number of proliferating cells had significantly increased (figure 5*b*,*h*) and dividing *foxj1a*:GFP^+^ ERGs around the central canal comprised almost 50% of all proliferating cells (47.05% ± 4.2%) (figure 5*b′*). At day 7 the number of proliferating cells had further increased, but the proportion of PCNA^+^ cells that expressed *foxj1a*:GFP was lower than at 3 dpi (23.5% ± 7%) (figure 5*c*,*c′*,*h*). The expansion of the ependymal area argued that new ependymal cells were being formed, but not all new cells expressed *foxj1a*:GFP. The transgene was also downregulated in new neurons as they left the ependymal zone and remained expressed only in CSF-cNs (electronic supplementary material, figure S8). At 14 dpi a large number of cells, mostly *foxj1a*:GFP-low or negative, was still proliferating at the injury epicentre (figure 5*e*,*e′*,*h*), but the number of dividing cells had decreased in adjacent regions (figure 5*d*,*d′*,*h*). By 30 dpi, the ependymal zone remained expanded, but the number of proliferating cells had considerably decreased, both far and close to the injury (figure 5*f–g′*,*h*). The dynamic behaviour of ERGs contrasted with the population of CSF-cNs in the ependymal region, which remained almost unchanged in response to injury (electronic supplementary material, figure S9).

These data reveal that the Foxj1a^+^ ERG population is sensitive to injury and initiates a proliferative programme that contributes to the expansion of the ependymal area. However, the presence of GFP-low or negative cells in the ependymal region raised the possibility that Foxj1a expression was not induced in new ERGs or that the reporter transgene was not responsive in these cells because it lacks the necessary regulatory elements, as observed in the embryonic neural tube.

### 2.6. Shh signalling activity induces Foxj1a expression during regeneration

**Figure 6.**
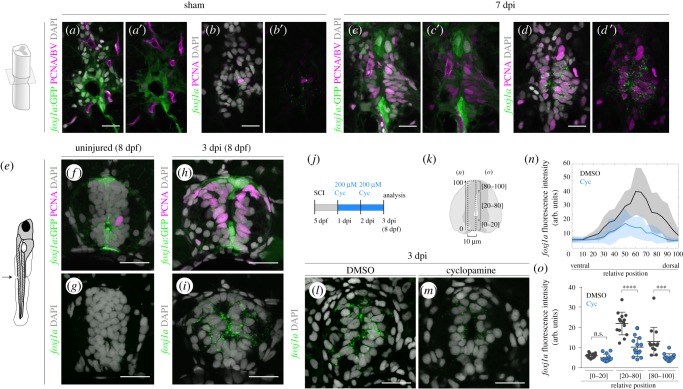
Endogenous Foxj1a expression is increased in response to injury in a Shh-dependent manner. (*a–d*′) Confocal images of transverse sections of *Tg(0.6foxj1a:GFP)* spinal cords immunostained against GFP (*a*,*c*) and FISH for *foxj1a* transcripts (*b*,*d*) in adjacent sections of sham-injury spinal cord (*a*,*b*) (*n* = 2) and 7 dpi spinal cord (*c*,*d*) (*n* = 8). The co-labelling with an antibody against PCNA (in magenta) shows that the majority of proliferative ERGs express *foxj1a* (*d*,*d*′) but not the *foxj1a*:GFP transgene (*c*,*c*′). BVs are also visible in (*a*) and (*c*) due to the expression of the transgene *Tg(flk1:mCherry)*. (*e*) Illustration of the site of spinal cord transection in 5 dpf larvae. (*f–i*) Representative confocal images of sections of 8 dpf *Tg(0.6foxj1a:GFP)* transgenic larvae, uninjured (*f*,*g*) (*n* = 8) or 3 dpi (*h*,*i*) (*n* = 10). Adjoining sections were either immunostained against GFP (green) and PCNA (magenta) (*f*,*h*) or processed with FISH for *foxj1a* transcripts (green) (*g*,*i*). (*j*) Schematic of the cyclopamine (Cyc) treatment experiments in injured larvae. (*k*) Schematic of the neural tube region selected to quantify the fluorescence levels of *foxj1a* transcripts plotted in (*n*) and (*o*). (*l*,*m*) Representative confocal images of transverse sections of 3 dpi spinal cords of larvae treated for 2 days with DMSO (*l*) or 200 µM cyclopamine (*m*) in the medium. The sections were collected 100 µm rostral to the injury site and *foxj1a* transcripts were detected with FISH. (*n*) Fluorescence intensity (f.i.) profile of *foxj1a* transcripts along the dorsoventral axis (relative to the size of the neural tube) show reduced *foxj1a* levels in Cyc-treated larvae (blue) (DMSO: *n* = 14; Cyc: *n* = 13) (a.u., arbitrary units). The line represents the mean f.i. and shaded regions correspond to the standard deviation intervals. (*o*) Quantification of the average *foxj1a* f.i. in the regions shown in (*k*). The mean and s.d. bars are also shown in (*n*,*o*) and the *p* values were calculated using two-tailed unpaired *t*-test (n.s., not significant; ***p < 0.001; ****p < 0.0001). DAPI-labelled nuclei are shown in grey. Scale bars, 20 µm.

To determine if *foxj1a*:GFP expression reproduced endogenous *foxj1a* in proliferating ERGs during regeneration, we compared the expression of the GFP reporter and the distribution of *foxj1a* transcripts in 7 dpi spinal cords. In sham-injury spinal cords, *foxj1a*:GFP labelled all cells around the central canal (figure 6*a*,*a′*). In adjoining sections, FISH for *foxj1a* revealed low levels of transcripts distributed around the central canal (figure 6*b*,*b′*). In injured spinal cords, the *foxj1a*:GFP reporter displayed high levels in the ventral and dorsal regions and low or absent expression in the middle region, where most PCNA^+^ cells were located (figure 6*c*,*c′*). The reverse pattern was seen for *foxj1a* transcripts: high transcript levels in the PCNA^+^ lateral cells and low signal in the dorsal and ventral regions (figure 6*d*,*d′*). These data show that Foxj1a is highly expressed by proliferating cells after an injury, confirming that Foxj1a^+^ ERGs actively participate in the repair of the lesioned spinal cord. In addition, these results reveal that the upregulation of *foxj1a* expression in response to injury is not replicated by the reporter transgene, suggesting that the injury-dependent expression of Foxj1a requires regulatory regions that are not present in the promoter sequence used in this transgenic line.

The activation of Foxj1a expression in new ependymal cells during regeneration raises the question of whether the injury-associated expression of Foxj1a is modulated by the signals used during development. Since in the developing neural tube Foxj1a expression in the floor plate is regulated by the Shh signalling pathway [16], we examined Foxj1a expression in lesioned spinal cords exposed to an inhibitor of the Shh pathway—cyclopamine. This experiment was carried out in larvae, which are more tractable than adults in terms of compound delivery and tissue penetrance [37–39]. We performed spinal cord transection in 5 dpf *Tg(0.6foxj1a:GFP)* larvae (figure 6*e*) and confirmed that *foxj1a* expression is also induced after spinal cord injury in 3 dpi larvae (figure 6*g*,*i*). Moreover, the distribution of *foxj1a* transcripts coincided with the accumulation of PCNA^+^ cells induced by the lesion (figure 6*f*,*h*), resembling the adult spinal cord.

We next examined the role of the Shh pathway in the upregulation of *foxj1a* in the injured tissue. Injured larvae were treated with 200 µM of cyclopamine or an equivalent volume of DMSO from day 1 to 3 post-injury (figure 6*j*). The concentration of cyclopamine used was sufficient to inhibit injury-induced proliferation in larvae (electronic supplementary material, figure S10). *Foxj1a* expression was detected by FISH in spinal cord sections of injured larvae and the fluorescence intensity of *foxj1a* transcripts was quantified in cells along the dorsoventral axis (figure 6*k*). In DMSO-treated larvae *foxj1a* transcripts were present at high levels in cells lining the lumen (figure 6*l*), with peak intensity at middle/dorsal positions (figure 6*n*,*o*). By contrast, the fluorescence intensity levels of *foxj1a* transcripts were dampened in larvae exposed to cyclopamine (figure 6*m*), with significantly lower levels in the middle/dorsal region when compared to the controls (figure 6*n*,*o*). This result suggests that Shh signalling activity is required for the injury-dependent expression of Foxj1a in ependymal cells.

Together, these data reveal that activation of the Shh signalling pathway after injury triggers upregulation of Foxj1a expression and promotes proliferation in the regenerating spinal cord.

## 3. Discussion

Ependymal cells were viewed simply as the lining of the central canal until the discovery of neural stem cells within this spinal cord population [8,10]. The identification of resident stem cells in the mammalian spinal cord led to a new therapeutic perspective in which ependymal cells could be used as an alternative to stem cell transplantation to help replace cell types lost after spinal cord injury [40]. However, further work is needed to understand how to overcome the astrocytic differentiation bias of these endogenous stem cells and to favour their differentiation into new neurons. Insight may be gained from the study of organisms that are able to spontaneously form new neurons and regain motor function after spinal cord injury, such as zebrafish [14].

Here, we identify Foxj1a as a general ependymal marker in zebrafish that can help follow the response of ependymal cells during spinal cord regeneration. Unlike other described molecular markers such as Sox2, GFAP and vimentin [41], Foxj1a is not restricted to subsets of ependymal cells. The expression of Foxj1a in the adult zebrafish spinal cord, together with the expression of other ependymal markers, reinforces the molecular similarities of stem/progenitor cells found in zebrafish and mammals. Nevertheless, when comparing our results obtained in zebrafish with the ones reported in mouse we could see differences in the ependymal region in terms of protein distribution (GFAP and vimentin) and radial glial morphology, which may be associated with differences in cellular function and could have an impact on their regenerative potential. We further show that Foxj1a is expressed by neurons that maintain attachments to the apical surface (CSF-cNs), indicating that Foxj1a labels not just ERGs but all cells contacting the central canal.

This study also reveals that Foxj1a is expressed in ependymal cells not only in the adult spinal cord but from the time of formation of the central canal in the embryonic neural tube. We show that Foxj1a is induced in the floor plate and roof plate before the initiation of lumen contraction. Foxj1a is also expressed in a subset of cells lining the lumen in the middle region of the neural tube. The different domains of Foxj1a-expressing cells later contribute to the ependymal region after the narrowing of the lumen. Although the expression of Foxj1 in the floor plate (in mouse, [27]) and in the roof plate (in chick, [28]) had been reported previously, here we show for the first time that Foxj1a expression is maintained in these two domains and in the previously unreported middle domain until the formation of the central canal. Moreover, we show that Foxj1a remains expressed in the ependymal region until adulthood.

Foxj1a is not only expressed by the cells that make up the ependymal zone, but is also required for the correct positioning of the central canal. In the absence of Foxj1a activity the dorsal to ventral zipping of the lumen is delayed resulting in a dorsally located canal. Foxj1a appears to be involved in the attenuation of the Shh-dependent mitogenic activity that is necessary for cells to exit the cell cycle and migrate away from the ventricular zone [33]. If this transition is affected, progenitor cells remain longer in the ventricular zone and the constriction of the primitive lumen is stalled. Although delayed, lumen contraction is completed in Foxj1a morphants. However, the final position of the central canal is moved dorsally, through a mechanism that is still unclear. The expanded Shh activity may affect the timing of differentiation along the dorsoventral axis, either by delaying the transition from proliferation to cell cycle exit or by changing the dorsoventral identity of progenitor cells, which influences their rate of differentiation [42]. If cells exit the lumen at the wrong time and/or order and the zipping of the lumen is not coordinated (for example, ventral and dorsal cells exiting at the same time), the central canal can end up in the wrong position. Nevertheless, the shifted ependymal region in the Foxj1a morphants displays a normal dorsoventral pattern, suggesting that ependymal cells originate from the correct progenitor domains. However, we cannot confirm that ependymal specification occurred without defects, since we did not test markers associated with mature ependymal cells. Therefore, further work is needed to assess if Foxj1a is required for the differentiation of ependymal cells, as shown in the mouse brain [43]. The mechanism through which Foxj1a dampens Shh signalling activity is also not known. The role of Foxj1 in the attenuation of Shh activity had been previously proposed in the chick neural tube [27], although the later effect on the position of the central canal had not been described. Foxj1 function in Shh pathway regulation is expected to involve changes in cilia structure that affect signal transduction downstream of Shh binding and make cells less responsive to Shh [27].

Foxj1a expression is also activated in new ependymal cells formed in response to injury, as had been suggested in embryos [17]. Injury-induced proliferation is first detected in the Foxj1a^+^ ependymal zone and results in the expansion of the ependymal layer. Many of the newly added ependymal cells continue to proliferate and reinforce the expression of Foxj1a, suggesting that these cells maintain a progenitor status. The upregulation of Foxj1a levels in the injured spinal cord depends on Shh activity, arguing that the regulation of Fox1a expression during regeneration relies on the same cues used during development. Shh expression is also increased in response to injury [23], but the trigger for the upregulation of Shh levels is still unknown. Shh activity also promotes proliferation in the regenerating spinal cord, and it will be of interest to investigate if Foxj1a function is required for the injury-induced proliferation or if, conversely, it is involved in the transition to differentiation in resemblance to its role during development.

The characterization of Foxj1a^+^ cells during spinal cord development and repair also revealed that the *foxj1a*:GFP reporter, which uses a 600 bp regulatory sequence to drive GFP expression [18], only partially reproduces the endogenous *foxj1a* expression. In the developing neural tube the reporter transgene fails to label Foxj1a^+^ cells in the middle region, and in the regenerating spinal cord the reporter is not induced in ependymal cells formed after injury. These results indicate that additional regulatory elements are necessary to fully reproduce endogenous Foxj1a expression, but further work is needed to identify these elements. It will be of particular interest to investigate if Foxj1a expression in response to injury requires dedicated regulatory sequences. Regeneration-specific enhancer elements were recently reported in zebrafish in the context of heart and caudal fin regeneration [44], and our data suggest that Foxj1a expression during spinal cord regeneration may also be regulated by specific enhancers.

A better understanding of the Foxj1a regulatory regions will also be important to develop new tools to follow ependymal cells after an injury. It is known that ependymal cells not only self-renew in response to injury, but also give rise to new neurons [14]. Given that Foxj1a is expressed by the majority of cells in the ependymal region, it is likely that Foxj1a^+^ cells are able to generate neurons. However, we were unable to identify the progeny of Foxj1a^+^ cells using the *Tg(0.6foxj1a:GFP)* transgenic line, due to the rapid downregulation of GFP expression in differentiated cells. To track the fate of Foxj1a descendants after they leave the ependymal region, it will be necessary to use a lineage-tracing approach, which will require a reliable Foxj1a promotor sequence. The lineage tracing of Foxj1a^+^ cells will help confirm if ERGs are multipotent (generating neurons, oligodendrocytes and astrocytes) and if all new neurons formed after injury arise from the ependymal population. In addition, the complete Foxj1a promoter can also be used to create cell ablation transgenic lines to evaluate the contribution of the ependymal population to the repair of the damaged tissue.

The role of Foxj1 as an ependymal marker is well established in amniotes [9,43]. Our study demonstrates that this role is conserved in zebrafish and shows that Foxj1a expression in ependymal cells is initiated in the developing spinal cord. Foxj1a also has an active role in the formation of the ependymal canal during development, through the modulation of the Shh-signalling pathway. Finally, this work shows that spinal cord injury induces the formation of new ependymal cells that contribute to tissue repair and Foxj1a expression is activated in these new cells through the use of specific regulatory elements. Thus, Foxj1 is a useful marker to help understand how similar populations of cells—ependymal cells in zebrafish and amniotes—display such strikingly different behaviours in response to injury that lead to regeneration in zebrafish and scarring in amniotes.

## 4. Material and methods

### 4.1. Fish husbandry and zebrafish lines

Adult and embryo zebrafish (*Danio rerio*) were maintained at 28°C in standard conditions. The zebrafish transgenic lines used in this study were previously reported: *Tg(0.6foxj1a:GFP)* [18], *Tg(flk1:mCherry)* [45] and *Tg(fli1:EGFP)^y1^* [46]. Procedures with zebrafish were approved by the Portuguese DGV (Direção Geral de Veterinária).

### 4.2. Morpholino oligonucleotide and mRNA microinjection

Foxj1a antisense MO ([16]; obtained from Gene Tools) and standard control MO (Gene Tools) were injected in *Tg(0.6foxj1a:GFP)* embryos at the one-cell stage. Each embryo was microinjected with a volume of 1.4 nl containing 2 ng of either Foxj1a or standard control MOs. Foxj1a MO-injected embryos were screened at 48 hpf to select only individuals displaying a curved body phenotype, which is indicative of problems in ciliogenesis [47]. To perform the rescue experiments, one-cell stage embryos were injected with 2 ng of *foxj1a* MO alone or together with either 500 pg or 1 ng of a MO-resistant form of the full length *foxj1a* mRNA.

### 4.3. Generation of a morpholino oligonucleotide-resistant full length *foxj1a* mRNA

The MO-binding region of the *foxj1a* coding sequence was altered by introducing silent mutations (forward primer: 5′-CGCGGGATCCGTCAGGACTATGTTAAGTATGTCTAGCATGGACCCCTGGCCGGA-3′; reverse primer: 5′-GCGCGCGGCCGCAAAGCAGAGTCTCGATTTTAC-3′). The transcription of the full length mRNA was performed using the SP6 mMESSAGE mMACHINE kit (ThermoFisher Scientific).

### 4.4. Generation of CRISPR gRNAs to target the *foxj1a* locus

CRISPR gRNAs targeting the sequence coding the fork head domain of the *foxj1a* gene were designed using the CRISPRscan algorithm for gRNA_FD1 [48] and the CRISPRdirect software for gRNA_FD2 [49]. The sgRNAs were generated according to Gagnon *et al*. [50]. Briefly, gene-specific oligonucleotides containing the T7 promoter, the target DNA sequence (underlined) and a complementary region (sgRNA_FD1: 5′-TAATACGACTCACTATAGGCAGGTGGGATCTGCGTGGGTTTTAGAGCTAGAAATAGCAAG-3′; sgRNA_FD2: 5′-TAATACGACTCACTATAGGCAGGTGGGATCTGCGTGGGTTTTAGAGCTAGAAATAGCAAG-3′) were annealed to an invariant oligonucleotide encoding the reverse complement of the tracrRNA tail (5′-AAAAGCACCGACTCGGTGCCACTTTTTCAAGTTGATAACGGACTAGCCTTATTTTAACTTGCTATTTCTAGCTCTAAAAC-3′) and T4 DNA polymerase (NEB #M0203S) was used to fill in the ssDNA overhangs. The double stranded sgRNA template was purified using QIAquick PCR Purification Kit (Qiagen #28104) and transcribed using MEGAshortscript™ T7 Transcription Kit (Ambion #AM1354). Finally, the sgRNAs were DNAse treated and purified with phenol:chloroform extraction followed by alcohol precipitation, according to the transcription kit. Cas9 protein was produced by the Weizmann Institute of Science, Israel as a purified batch at 1 mg ml^−1^ in 20 mM Tris pH 8.0, 10 mM MgCl_2_, 0.2 M KCl. For the gRNA/Cas9 microinjections, the two sgRNAs (FD1 and FD2) were mixed with Cas9 protein (360 ng µl^−1^ per sgRNA and 800 ng µl^−1^ of Cas9 protein) and incubated at 37°C for 5 min to allow for the sgRNA/Cas9 complexes to form. Embryos at one-cell stage were injected with 1.4 nl of this solution.

### 4.5. Spinal cord injury in adult zebrafish

Adult zebrafish of either sex (4–12 month-old) were anaesthetized by immersion in 0.016% tricaine (MS222, Sigma) in tank water. A longitudinal incision was made on the side of anaesthetized zebrafish, midway between the base of the skull and the dorsal fin. After exposing the vertebral column, the spinal cord was compressed dorsoventrally using forceps (Dumont #55, FST). The wound was sealed with Vetbond (3M) and the fish allowed to recover in individual tanks. In control fish a sham injury was performed by making an incision at the side of the animal but leaving the spinal cord intact before sealing the wound.

### 4.6. Spinal cord injury and cyclopamine treatment in zebrafish larvae

The protocol to perform spinal cord injury in zebrafish larvae was previously described [38]. Transgenic larvae (*Tg(0.6foxj1a:GFP)*) at 5 dpf were anaesthetized in 0.016% tricaine in embryo medium and placed on their side in a drop of medium in a Petri dish. The injury was performed at the level of the anal pore using a 20G × 1″ syringe needle (Terumo) to transect the region dorsal to the notochord that contains the spinal cord. Injured larvae were carefully transferred to fresh embryo medium and maintained at 28°C until the time of analysis.

To inhibit the Shh signalling pathway in injured larvae, cyclopamine (Sigma, C4116) was added at a concentration of 200 µM to the medium 1 day after the injury and fresh medium with cyclopamine was added again at 2 dpi. A control group of injured larvae was treated with an equivalent volume of DMSO (cyclopamine vehicle). The drug-treated larvae were kept in the dark at 28°C until the time of analysis.

### 4.7. Histology

The vertebral column of adult zebrafish was dissected and fixed in 4% paraformaldehyde (PFA) at 4°C overnight. After fixation the spinal cord was isolated from the vertebral column. Embryos/larvae collected at different developmental stages were also fixed in 4% PFA at 4°C overnight. The samples were washed for 3 × 5 min in PBS and equilibrated in 15% sucrose/0.12 M phosphate buffer (PB) pH 7.2 solution at 4°C overnight, embedded in 7.5% gelatin/15% sucrose/0.12 M PB pH 7.2 at 42°C and frozen. The samples were cryosectioned in 14 µm-thick slices and processed for immunohistochemistry and *in situ* hybridization.

### 4.8. Transmission electron microscopy

The vertebral column of adult zebrafish was dissected and the spinal cord isolated from the vertebrae before fixation overnight at 4°C in 0.1 M sodium cacodylate buffer, pH 7.4, containing 2.5% (v/v) glutaraldehyde. The samples were post-fixed in 1% (aqueous) osmium tetroxide for 1 h and contrasted in 1% (aqueous) uranyl acetate for 30 min. Dehydration was made using ethanol gradient (50–70–95–100%) and infiltration was aided using propylene oxide and a mixture (1 : 1) of propylene oxide and EPON 812 resin (EMS). Samples were embedded in EPON resin and hardened at 60°C for 36 h. Sections were obtained using an ultramicrotome Reichert Supernova (Leica Microsystems), and semi-thin sections (500 nm) were stained with toluidine blue for light microscope evaluation. Ultra-thin sections (70 nm) were collected in Formvar (AGAR Scientific) coated copper slot grids, and counter-stained with uranyl acetate and lead citrate (Reynold recipe), and screened in a Hitachi H-7650 transmission electron microscope at 100 kV acceleration.

### 4.9. Immunohistochemistry

To perform immunostaining in sections the gelatin was removed from the cryosections using PBS heated to 42°C (4 × 5 min washes). After incubation with a blocking solution (1% bovine serum albumin in PBS/0.01% Triton X-100 (PBSTx)) for 1 h at room temperature (RT), the sections were incubated in primary antibody solution at 4°C overnight. After 5 × 5 min washes in PBSTx, the sections were incubated with the secondary antibody (1 : 1000 in blocking solution) and 1 µg ml^−1^ DAPI (Sigma, D9564) for 2 h at RT. Details of the primary and secondary antibodies used are described in the electronic supplementary material, tables S1 and S2. After incubation with the secondary antibodies, the sections were washed in PBS and mounted in Mowiol mounting medium. Antigen retrieval was performed for the anti-PCNA antibody by placing the slides in 10 mM sodium citrate pH 6/0.1% Triton X-100 and heating in a microwave at sub-boiling temperature for 5 min before proceeding with gelatin removal and immunostaining.

### 4.10. *In situ* hybridization

*In situ* hybridization (ISH) was performed in sections using probes against *foxj1a* [51], *pkd2l1* (kindly provided by Mónica Roxo-Rosa) or *patched2* [34]. Digoxigenin or fluorescein (Fluo)-labelled probes were transcribed using T7 or SP6 RNA polymerase (Roche). The ISH protocol followed was adapted from a described protocol [52] and is detailed in the electronic supplementary material. In sections that were processed with a colorimetric reaction, the probe was developed using BM Purple AP substrate (Roche, 11442074001). In sections developed with a fluorescent reaction (FISH), the TSA Plus TMR/Fluorescein system (PerkinElmer, NEL756001KT) was used instead, following the manufacturer's protocol. Nuclei were counterstained with 1 µg ml^−1^ DAPI and the slides were mounted in Mowiol medium. In some cases, the FISH was followed by immunostaining against GFP or PCNA. In these sections, the development of the probe with the TSA plus system was followed by the immunohistochemistry procedure described above.

### 4.11. Imaging

Immunostained and FISH sections were imaged using Zeiss LSM 710 or LSM 880 confocal microscopes with Plan-Neofluor 40×/1.3 Oil or Plan-Apochromat 63×/1.4 Oil objectives. Each image is a maximum intensity projection of a *z*-stack acquired from the 14 µm cryosection. Images of the neural tube region in transverse sections of larvae were acquired at the level of somites 5–10. The images of ISH sections developed with colorimetric reaction were acquired using a Leica DM2500 brightfield microscope with a HCX PL FLUOTAR 100×/1.3 Oil objective. The processing of acquired images was performed using the image analysis software Fiji [53] and Adobe Photoshop and Illustrator were used for assembly of figures.

### 4.12. Quantification of lumen closure

The positions of the dorsal and ventral points of the lumen and the ventral and dorsal edges of the neural tube (floor plate and roof plate, respectively) were quantified manually using Fiji. Matlab was used to register the positions in each neural tube to the centre of the lumen and to plot the grouped positions according to stage (hpf) and condition (Foxj1a MO versus Standard MO). GraphPad Prism 5 was used to generate the scatter plot of the size of the lumen and ventral and dorsal regions and to perform statistical analysis between conditions.

### 4.13. Quantification of cell number and positions

The number of phospho-histone H3^+^ cells per section was quantified manually and obtained data were plotted and analysed statistically using GraphPad Prism v. 5.

The number and position of PCNA^+^ (or Hu^+^), *foxj1a*:GFP^+^ and DAPI^+^ cells in the injured spinal cords were quantified using the 3D Objects Counter plugin available in Fiji [54]. Briefly, a maximum projection image was generated from three optical sections with 1 µm between sections. The image of the DAPI channel was then used to automatically segment individual nuclei using the plugin. In nuclei that overlapped and were not segmented automatically, the boundary between nuclei was defined manually. The segmentation mask that identified individual objects (nuclei) in the DAPI channel was then used to extract the fluorescence intensity in the other two channels (GFP and PCNA/Hu). The plugin output included the position of each nucleus and the average fluorescence intensity in the GFP and PCNA/Hu channels. We defined that a nucleus was positive for a specific channel if the average intensity was above the background levels (measured manually). Matlab was used to analyse the obtained data and to generate scatter plots of cell positions for each time-point after injury (composite maps).

GraphPad Prism 5 was used to plot the total number of different cell types per section, to compare between different conditions and different time-points post-injury and to perform statistical analysis.

### 4.14. Quantification of fluorescence intensity of *foxj1a* and *shh* transcripts

Images of sections labelled by FISH with a probe against *foxj1a* or *shh* were used to measure the profiles of the fluorescence intensity of *foxj1a* or *shh* transcripts along the dorsoventral axis. A 10 µm-wide box was placed on one of the sides of the lumen from the ventral to the dorsal edges of the neural tube and the dorsoventral profile of *foxj1a* or *shh* fluorescence intensity (in arbitrary units, a.u.) was measured inside the box using the Plot Profile plugin in Fiji. Matlab was used to normalize the dorsoventral positions to the size of the neural tube and to bin the raw fluorescence intensity values into 5% intervals relative to the total size of the neural tube. The binned fluorescence intensity was averaged between individuals and plotted as a function of the relative dorsoventral position. Matlab was also used to quantify the *foxj1a* or *shh* fluorescence intensity in different dorsoventral regions of the neural tube. GraphPad Prism 5 was used to plot the average *foxj1a* fluorescence intensity in each region, to compare between different conditions (DMSO versus Cyc) and perform statistical analysis.

## Supplementary Material

Ribeiro_etal_2017_Sup_Files

## Data accessibility

The datasets supporting this article have been uploaded as part of the electronic supplementary material.
