# Correction to ‘Foxj1a is expressed in ependymal precursors, controls central canal position and is activated in new ependymal cells during regeneration in zebrafish’

**DOI:** 10.1098/rsob.180082

**Published:** 2018-05-30

**Authors:** Ana Ribeiro, Joana F. Monteiro, Ana C. Certal, Ana M. Cristovão, Leonor Saúde

*Open Biol.* 7, 170139. (Published online 21 November 2017). (doi:10.1098/rsob.170139).

A correction is required to the sequence of sgRNA_FD2 in [1].

The correct sequence is as follows:

sgRNA_FD2:

5′-TAATACGACTCACTATATTCCAGAAGCCGCCTTTGCCCGGGTTTTAGAGCTAGAAATAGCAAG-3′

All the authors gave their consent to this correction.
