# Supplementary material for: Foxj1a is expressed in ependymal precursors, controls central canal position and is activated in new ependymal cells during regeneration in zebrafish
Source: Open Biol. 2017 Nov 22;7(11):170139. doi: 10.1098/rsob.170139 (PMC5717339; doi:10.1098/rsob.170139)
Supplement: Ribeiro_etal_2017_Sup_Files [file rsob170139supp1.pdf]

Supplementary Figures

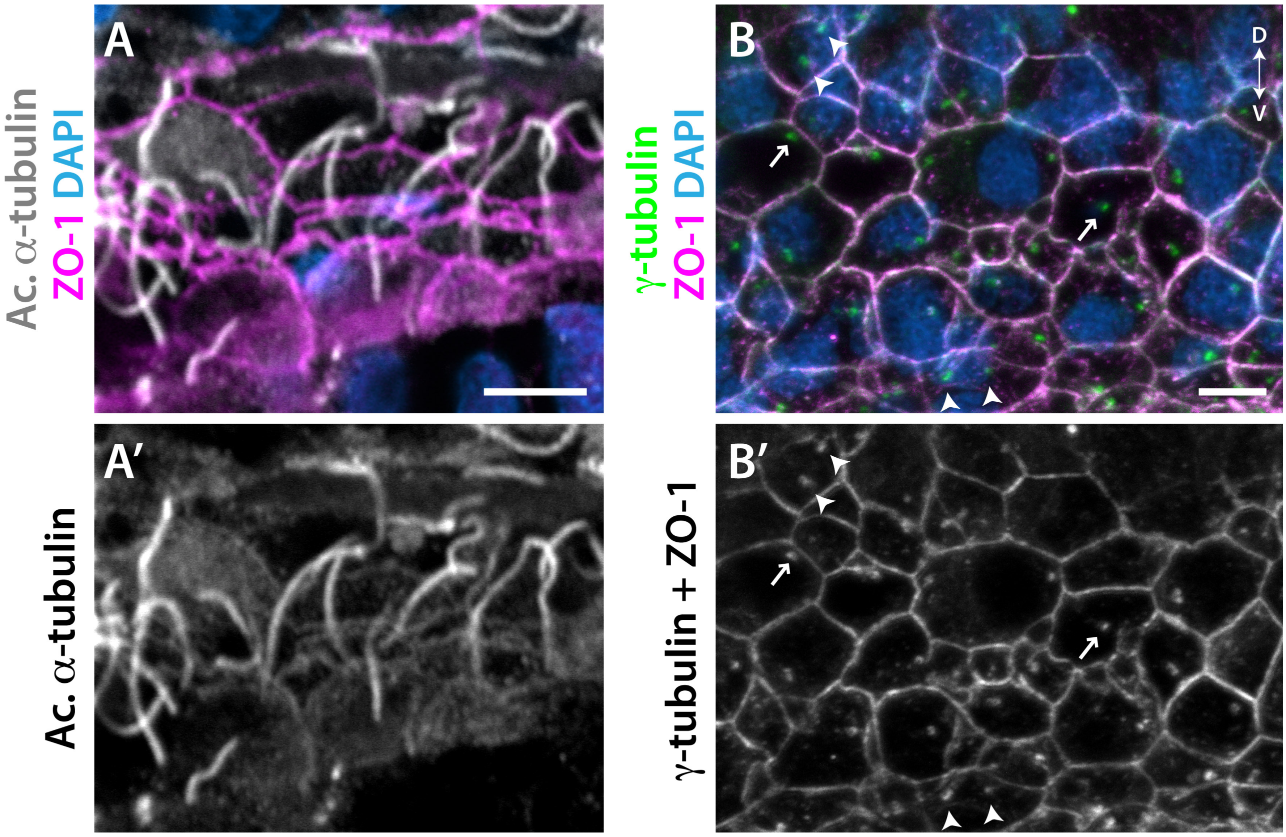

Supplementary Figure 1.

Ependymo-radial glial cells display one or two cilia in the zebrafish adult spinal cord.
(A,A') Confocal stack projection of a spinal cord sagittal section at the level of the ependymal region, immunostained with acetylated α-tubulin (grey) to label cilia and ZO-1 (magenta) to label the apical edges of the cells surrounding the central canal. Cilia are arranged as isolated structures, not as bundles. **(B,B')** Confocal image of the lateral wall of the ependymal region in a sagittal section of the adult spinal cord, with the apical edges of cells labelled by ZO-1 (magenta) and the basal bodies labelled by ɣ-tubulin (green). Ependymal cells display either one (arrow) or two (arrowhead) cilia. The dorsal (D) and ventral (V) orientation is also shown. DAPI-labelled nuclei are shown in blue. Scale bars: 5 µm.

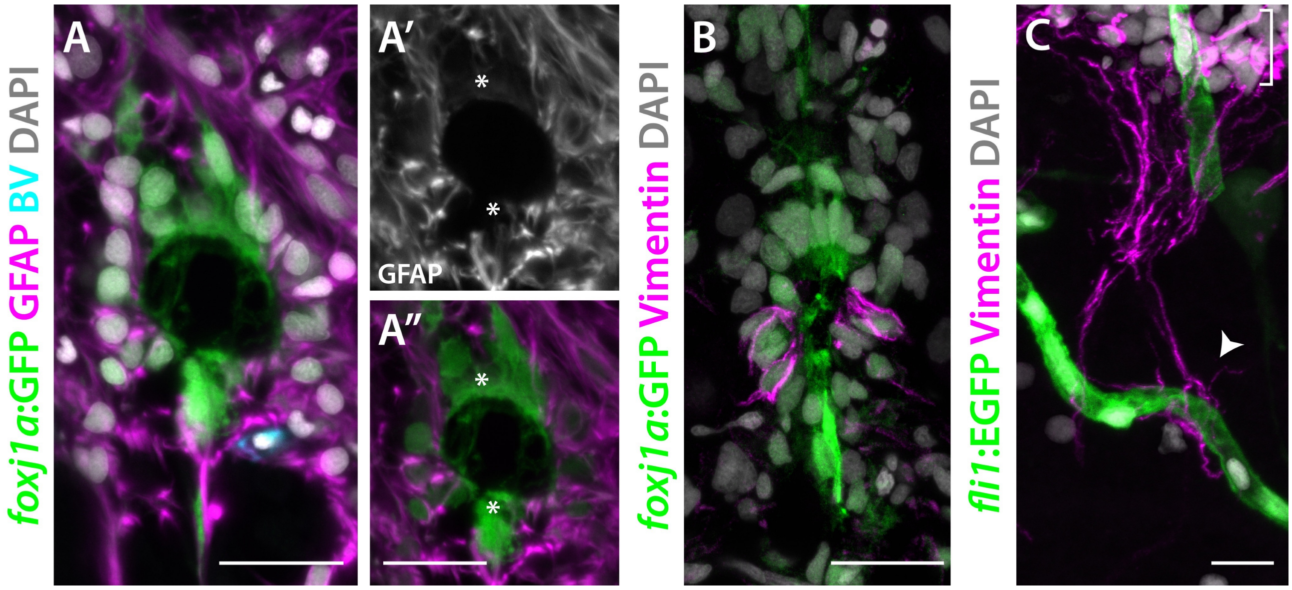

Supplementary Figure 2.

Molecular markers associated with radial glial character are expressed in only a subset of ependymal cells.

(A-A'') Confocal stack projection of a spinal cord transverse section of *Tg(0.6foxj1a:GFP); Tg(flk1:mCherry)* transgenic zebrafish, immunostained with GFAP (magenta), a marker of radial glial cells. GFAP is not expressed by all foxj1a:GFP$^+$ cells (asterisks in A' and A''). GFAP signal is detected around blood vessels (BV) located close to the ependymal canal and GFAP$^+$ cells possibly incorporate the vascular niche. **(B)** Confocal image of a transverse section of a *Tg(0.6foxj1a:GFP)* spinal cord, labelled with an antibody against Vimentin (magenta), another radial glial marker. Again, only a subset of cells in the medial-ventral region express Vimentin. **(C)** Confocal image of a sagittal section of a *Tg(fli1:GFP)* spinal cord, where blood vessels are labelled in green. Vimentin$^+$ cells (magenta) in the ependymal layer (bracket) extend projections that contact and encircle blood vessels (arrowhead). The morphology of Vimentin$^+$ cells and their interaction with blood vessels suggest that they are tanycytes. DAPI-labelled nuclei are shown in grey. Scale bars: 20 μm in A-B and 10 μm in C.

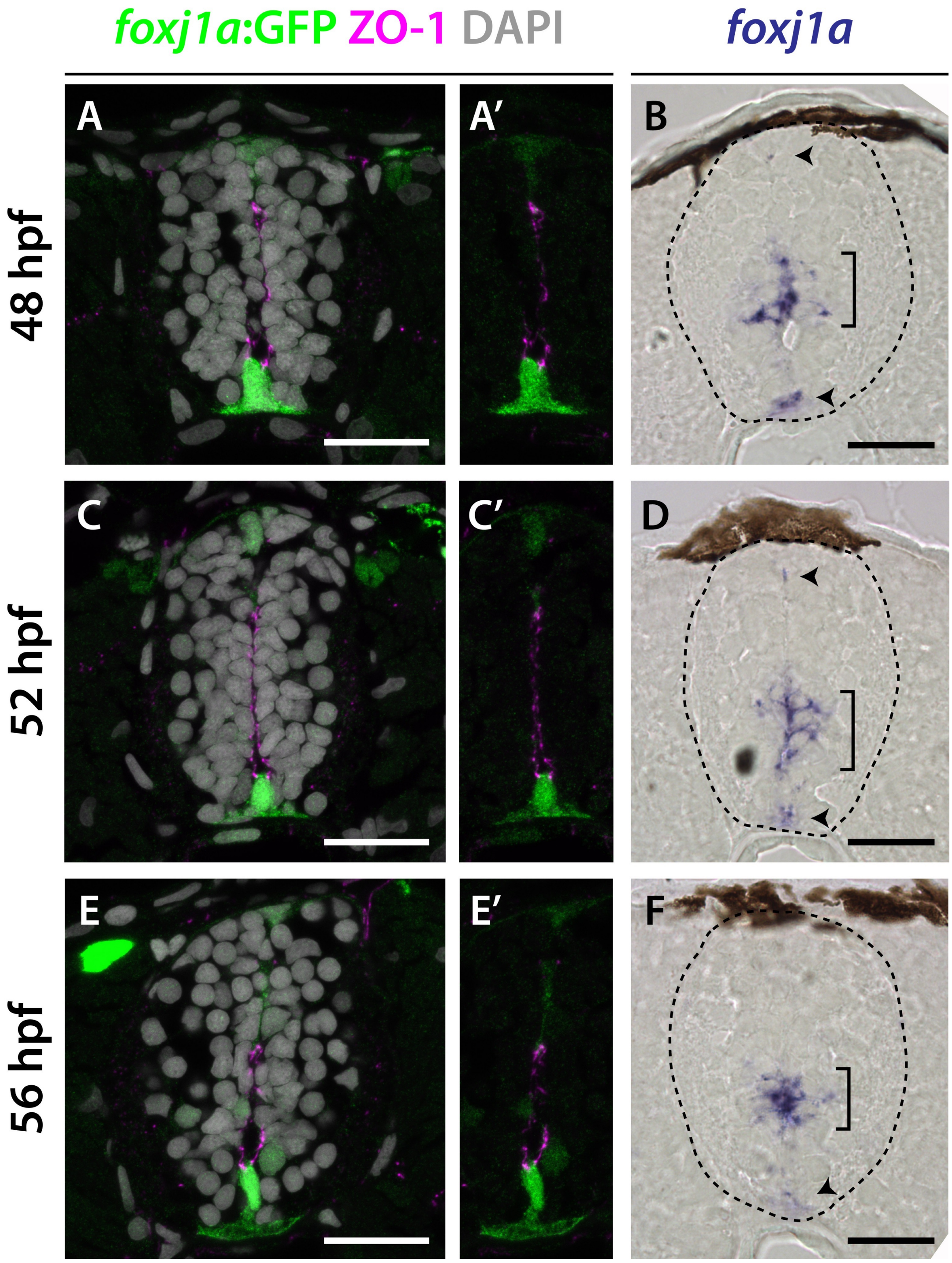

Supplementary Figure 3.

foxj1a morphants display body curvature typical of ciliogenic defects.

(A,A′,C,C′,E,E′) Representative images of *Tg(0.6foxj1a:GFP)* transgenic embryos at 48, 52 and 56 hours-post fertilization (hpf). The edge of the lumen is identified by ZO-1 immunostaining (magenta). **(B,D,F)** *foxj1a* colorimetric in situ hybridisation in transverse sections adjacent to to the sections shown in **(A,C,E)**. *foxj1a* expression in the roof plate and floor plate is identified with arrowheads and the middle domain is identified by a bracket. Scale bars: 20 μm.

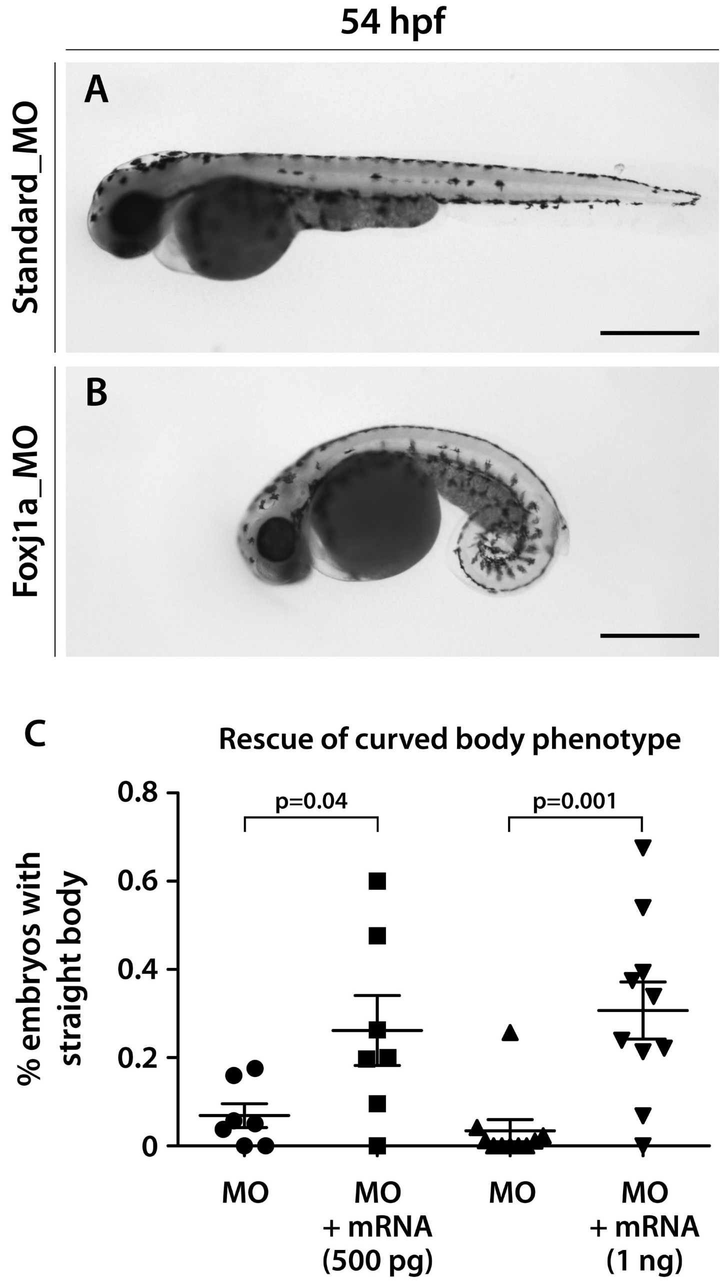

Supplementary Figure 4.

***foxj1a* morphants display body curvature typical of ciliogenic defects that are rescued by co-injection with *foxj1a* mRNA.**

(A,B) Representative images of *Tg(0.6foxj1a:GFP)* transgenic 54 hours-post fertilization (hpf) embryos injected with Standard Morpholino (MO) **(A)** or *foxj1a* MO **(B)** at one-cell stage. **(C)** Quantification of the percentage of embryos with straight body per batch after microinjection with *foxj1a* MO alone or with 500 pg or 1 ng of *foxj1a* mRNA (n=7 microinjections for 500 pg; n=10 microinjections for 1 ng). Scale bars: 500 µm.

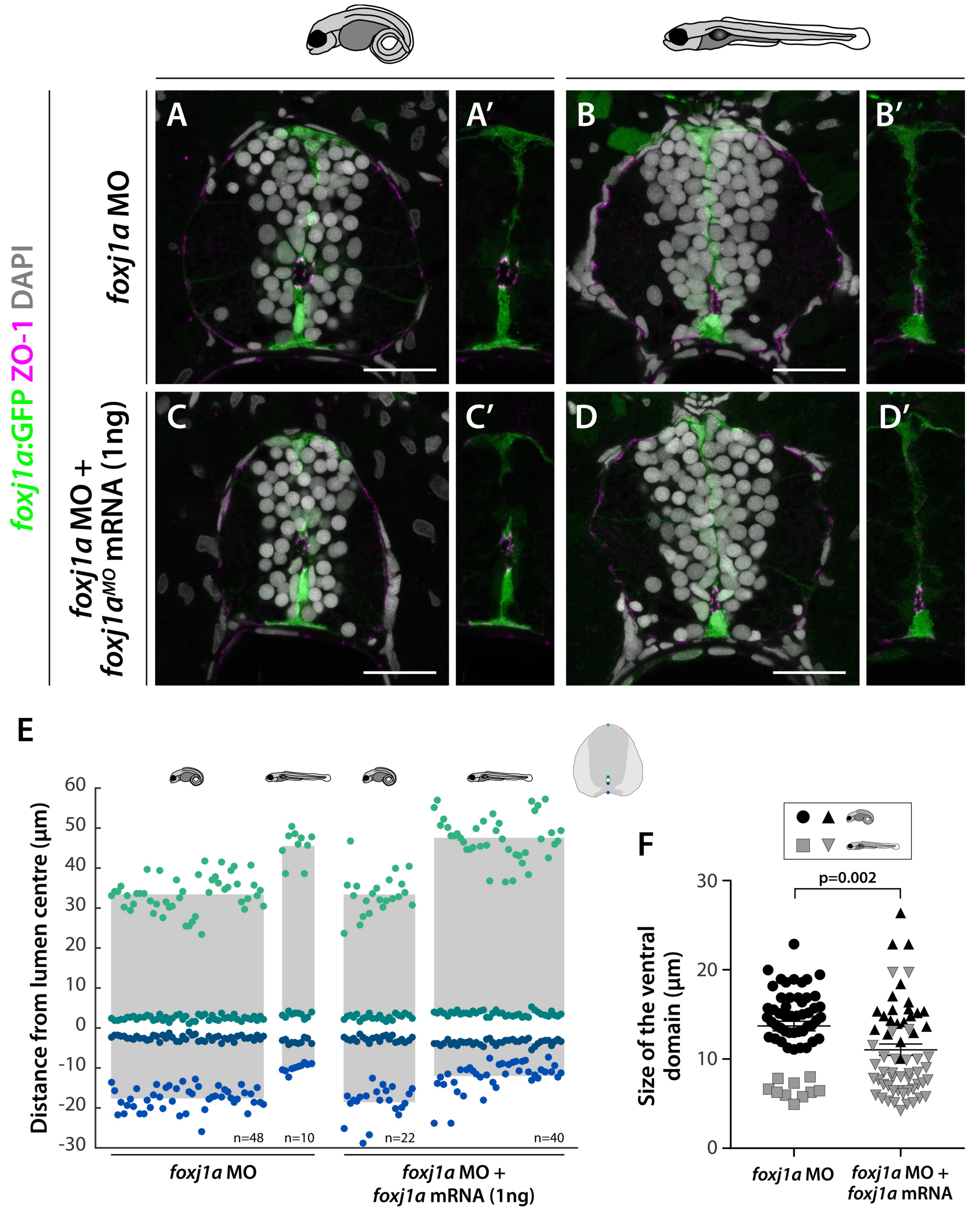

Supplementary Figure 5.

Rescue of *foxj1a* MO curved body phenotype in embryos co-injected with *foxj1a* mRNA is accompanied by the rescue of the position of the central canal.

(A-D′) Representative images of *Tg(0.6foxj1a:GFP)* transgenic larvae with 5 days-post fertilization injected with *foxj1a* MO alone **(A-B′)** or *foxj1a* MO injected together with 1 ng of *foxj1a* mRNA **(C-D′)**. The lumen is highlighted by ZO-1 expression in magenta. **(E)** Quantification of the positions of the floor plate, lumen and roof plate (normalized to the middle point of the lumen) in larvae injected with *foxj1a* MO alone or *foxj1a* MO injected together with 1 ng of *foxj1a* mRNA. Shaded area represents the average size of the ventral and dorsal regions. Sample number is shown in the graph and includes data from 3 independent experiments. **(F)** Quantification of the size of the ventral region. Each point represents 1 individual and the mean and S.D. bars are also shown. P value calculated using two-tailed unpaired t-test. Scale bars: 20 µm.

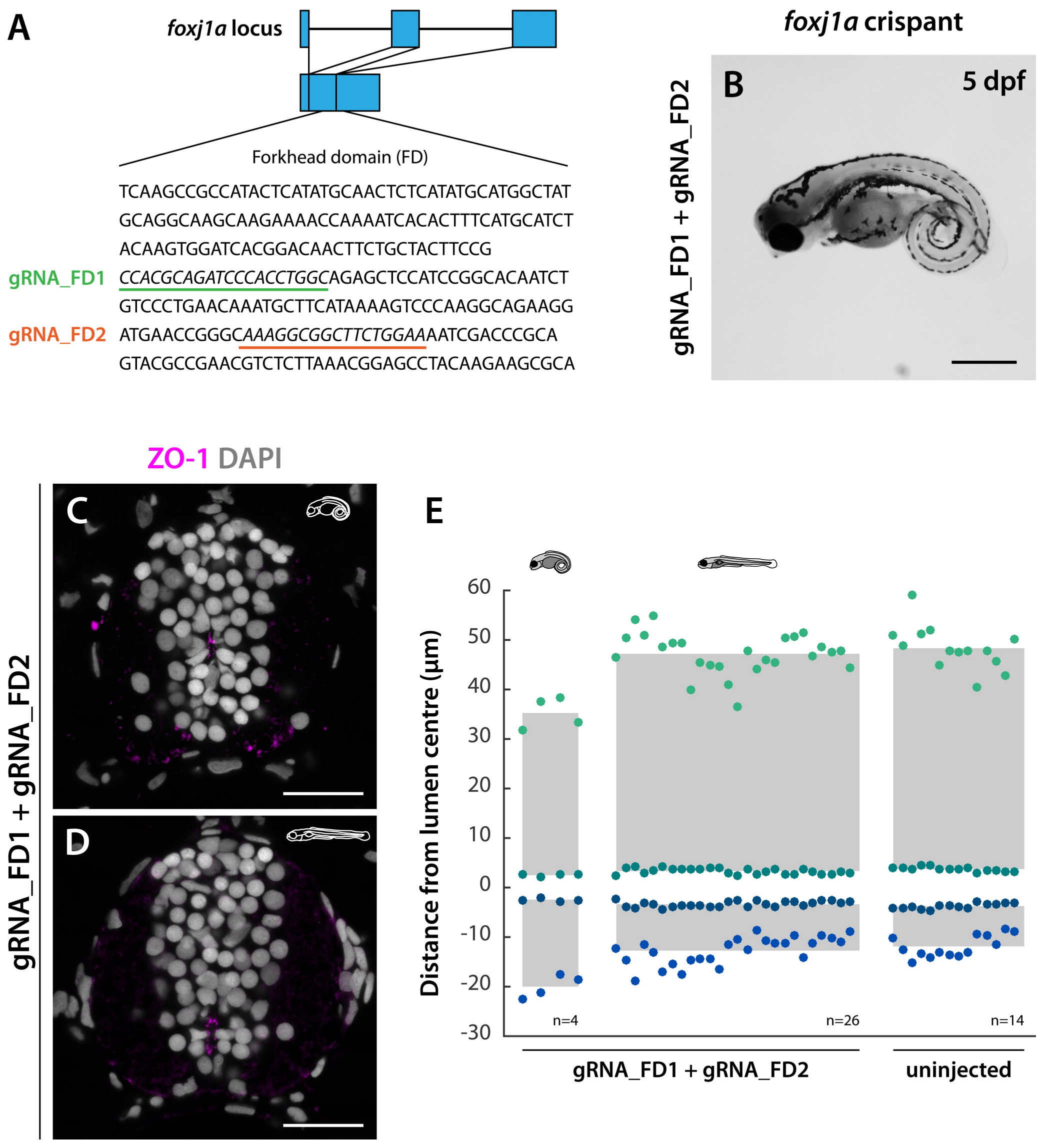

Supplementary Figure 6.

***foxj1a* crispants display curved body phenotype and dorsalized position of the central canal similar to *foxj1a* morphants.**

(A) Design of guide RNAs (gRNAs) targeting two different regions of the sequence coding for the Forkhead domain in the *foxj1a* locus. (**B**) Image of a larva with 5 days-post fertilization (dpf) injected with Cas9/gRNA_FD1 and Cas9/gRNA_FD2 complexes at one-cell stage showing a curved body. Larvae with curved body (crispants) were very rare among injected larvae (<0.8%). **(C,D)** Confocal images of sections of 5 dpf larvae injected with Cas9 protein/gRNAs complexes, immunostained for ZO-1 to label the central canal. In larvae with curved body **(C)** the central canal is located in a more dorsal position than in larvae with straight body **(D)**. **(E)** Quantification of the positions of the floor plate, lumen and roof plate (normalized to the middle point of the lumen) in larvae injected with Cas9 protein/gRNAs complexes (curved vs. straight body) and in uninjected larvae. Shaded area represents the average size of the ventral and dorsal regions. Sample number is shown in the graph. Scale bars: 500 μm (B) and 20 μm (C,D).

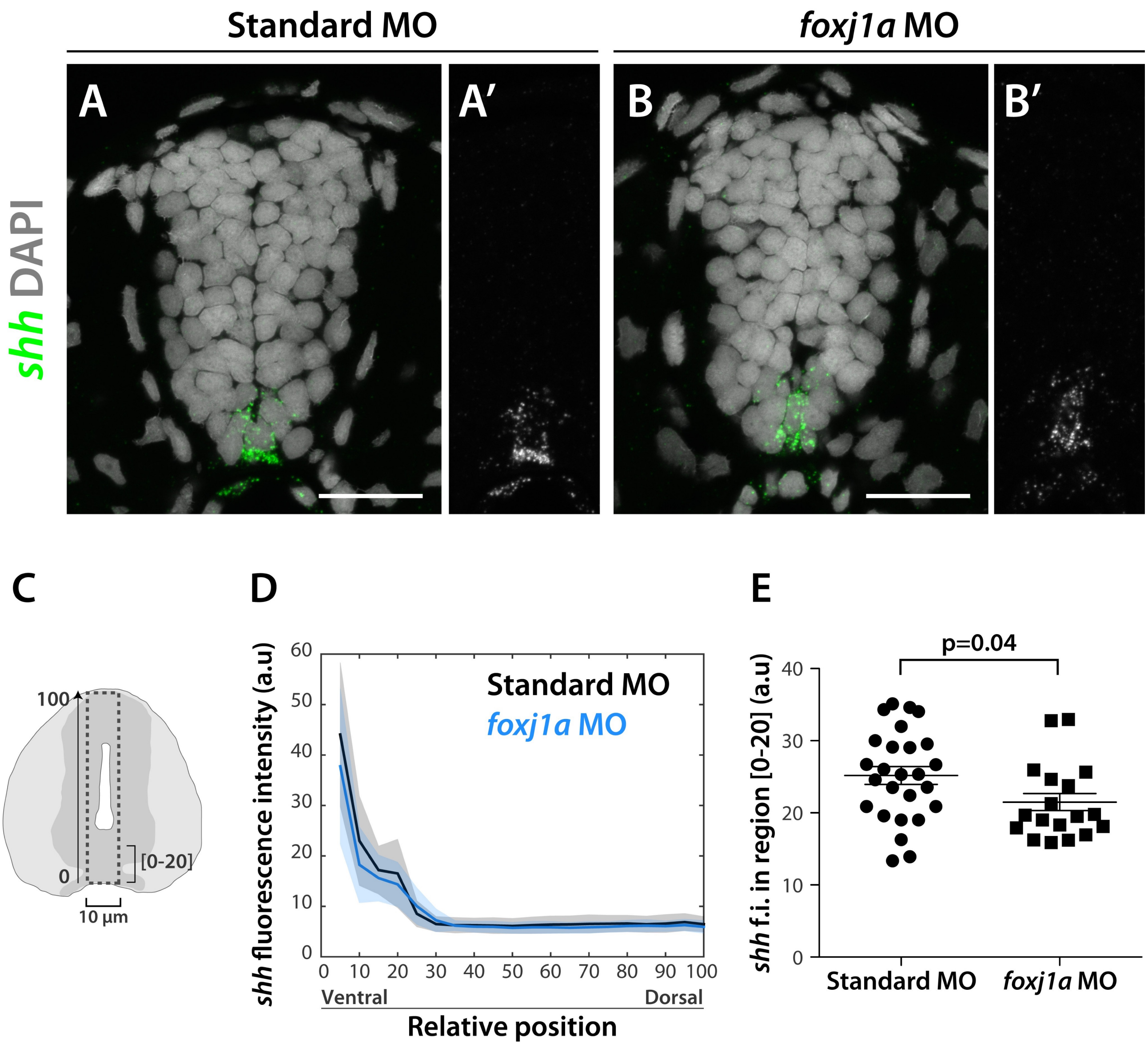

Supplementary Figure 7.

***shh* expression is unaltered in *foxj1a* morphants.**

(A-B') Representative images *shh* FISH in sections of 54 hours-post fertilization embryos injected with Standard Morpholino (MO) **(A-A')** or *foxj1a* MO **(B-B')**. **(C)** Schematic of the neural tube region selected to quantify the fluorescence levels of *shh* transcripts plotted in **(D)** and **(E)**. **(D)** Fluorescence intensity (f.i.) profile of *shh* transcripts along the DV axis (positions normalised to the size of the neural tube) in Standard MO and *foxj1a* MO embryos. (a.u., arbitrary units) The line represents the mean f.i. and shaded regions correspond to the standard deviation intervals. **(E)** Quantification of the average *shh* f.i. in the ventral region ([0-20] - most ventral 20% of the total neural tube size). P value calculated using two-tailed unpaired t-test. Scale bars: 20 µm.

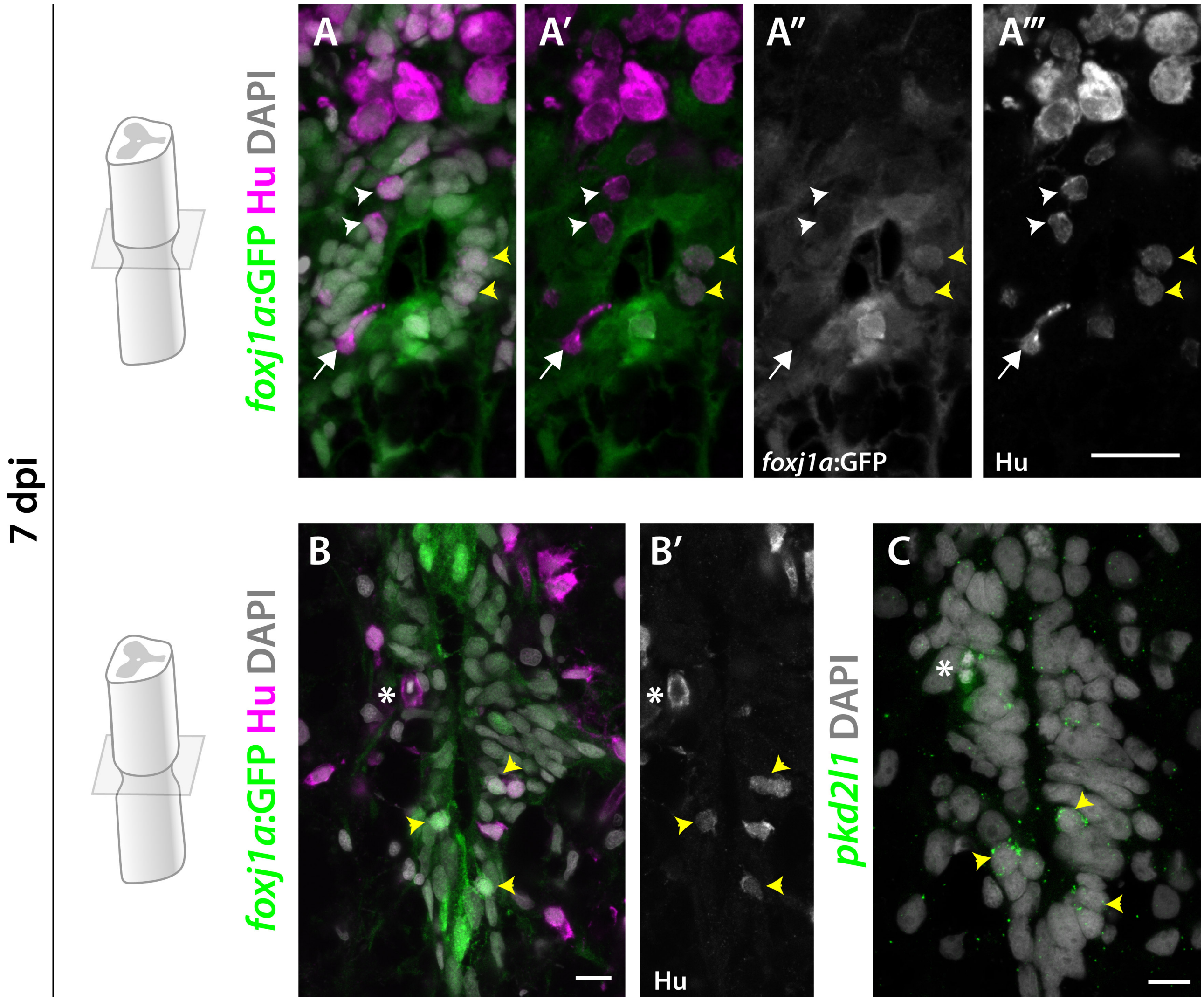

Supplementary Figure 8.

Foxj1a expression is downregulated in differentiating neurons formed after injury.

(A-A‴) 7 dpi spinal cord transverse section at a distance of 350 μm from the injury centre, showing differentiated neurons that lack *foxj1a*:GFP expression and are detaching (Hu$^+$,white arrow) or have detached (Hu$^+$,white arrowheads) from the apical surface. CSF-cNs (Hu$^+$/ *foxj1a*:GFP$^+$) are identified by yellow arrowheads. New neurons migrating away from the ependymal region rapidly downregulate GFP expression. **(B,B′)** Transverse section close to the injury in a 7 dpi spinal cord, showing Hu$^+$/*foxj1a*:GFP$^+$ cells with CSF-cN morphology (yellow arrowheads). **(C)** FISH for *pkd2l1* in a section adjacent to the section in **(B)**, with labelled CSF-cNs (yellow arrowheads) at similar positions to the cells in **(B)**. A contiguous blood vessel is detected in both sections (asterisks). CSF-contacting neurons are the only neurons in the ependymal region that are labelled by *foxj1a*:GFP. DAPI-labelled nuclei are shown in grey. Scale bars: 20 μm in (A-A‴); 10 μm in (B,C).

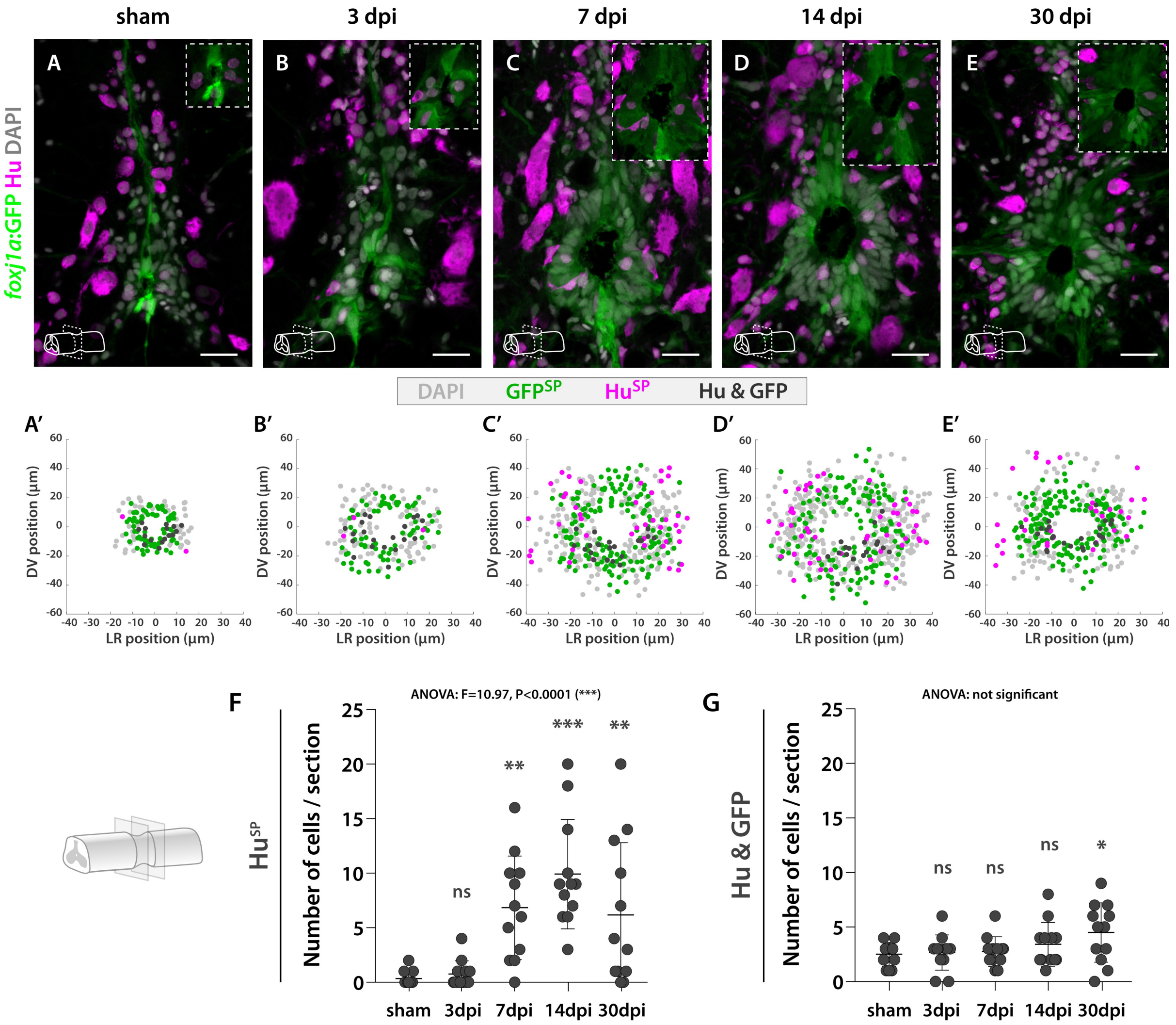

Supplementary Figure 9.

CSF-contacting neurons are not affected by spinal cord injury.

(A-E) Representative confocal images of transverse sections of spinal cords of *Tg(0.6foxj1a:GFP)* transgenic zebrafish. **(A)** Sham injury. **(B-E)** 3 to 30 days after spinal cord injury (dpi, days post-injury). Neurons (in magenta) were labelled using an antibody against HuC/D and cells expressing the GFP reporter are shown in green. **(A'-E')** Composite maps of cell positions quantified in transverse section images of six different spinal cords for each time point. CSF-cNs (dark grey) remain ventrally located over the course of regeneration. DV - dorsal/ventral; LR - left/right. **(F,G)** Quantification of the number of Hu single positive cells **(F)** and Hu/*foxj1a*:GFP double positive cells **(G)** per section. The number of Hu/*foxj1a*:GFP cells, corresponding to CSF-cNs, remains unchanged in response to injury. Each circle represents one section at 350 μm rostral or caudal from the injury centre (two sections per spinal cord and six fish per time point). The mean and S.D. bars are also shown and a one-way ANOVA F test was performed between groups, followed by a Dunnett test to compare every mean to the control mean (ns, not significant; *p<0.05; **p<0.01; ***p<0.001). DAPI-labelled nuclei are shown in grey. Scale bars: 20 μm in (A-E).

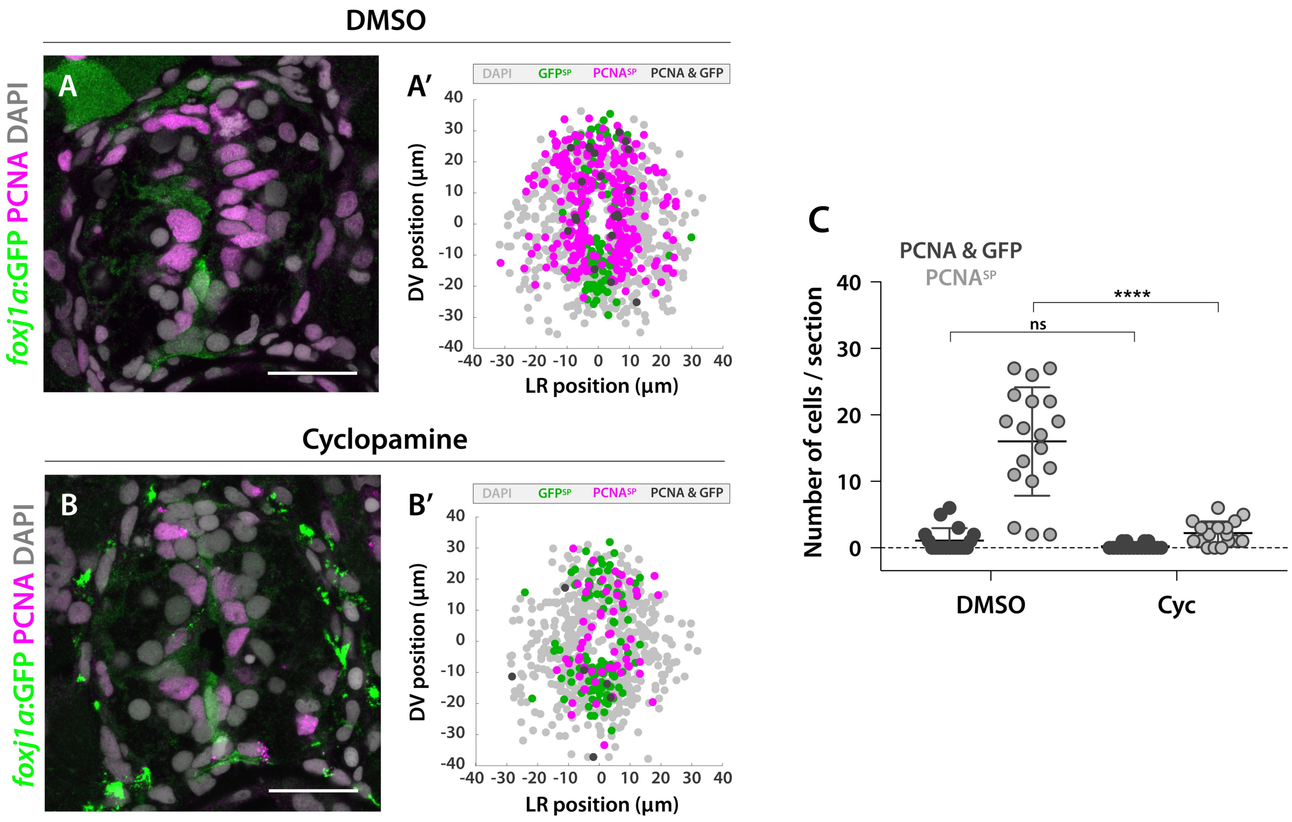

Supplementary Figure 10.

Inhibition of Shh signalling restricts injury-induced proliferation in larval spinal cords.

(A,B) Representative confocal images of transverse sections of 3 days post-injury (dpi) spinal cords of *Tg(0.6foxj1a:GFP)* transgenic larvae treated for 2 days with DMSO **(A)** or 200 µM Cyclopamine **(B)** in the medium. The sections were collected 100 µm rostral to the injury site and immunostained against GFP (green) and PCNA (magenta). **(A′,B′)** Composite maps of cell positions quantified in transverse section images of several spinal cords for each condition (n=18/condition, 3 independent experiments). DV - dorsal/ventral; LR - left/right; SP - single positive. **(C)** Quantification of the number of $PCNA^{SP}$ (light grey) and $PCNA^{+}/GFP^{+}$ (dark grey) cells per section. The mean and S.D. bars are also shown and the P values were calculated using two-tailed unpaired t-test (ns, not significant; ****p<0.0001). DAPI-labelled nuclei are shown in grey. Scale bars: 20 µm.

Supplementary Experimental Procedures

Supplementary Table 1. Primary antibodies used.

Antigen	Host	Dilution	Source/Reference
GFP	Rabbit	1:2000	Abcam/ab290
GFP	Chicken	1:500	Aves Labs/GFP-1020
Acetylated α-tubulin	Mouse	1:500	Sigma/T7451
Acetylated γ-tubulin	Mouse	1:200	Sigma/T5326
GFAP	Mouse	1:100	ZIRC/zrf-1
Vimentin	Mouse	1:50	DSHB/3CB2
HuC/D	Mouse	1:500	Life Technologies/A21271
ZO-1	Mouse	1:500	ThermoFisher Scientific/ 339194
Phospho-Histone H3 (pHH3)	Mouse	1:500	Millipore/1545035
Proliferating Cell Nuclear Antigen (PCNA)	Mouse	1:500	Dakocytomation/M 0879

Supplementary Table 2. Secondary antibodies used.

Against	Host	Fluorophore	Source/Reference
Rabbit	Goat	Alexa Fluor 488	ThermoFisher Scientific/A11008
Chicken	Goat	Alexa Fluor 488	ThermoFisher Scientific/A11039
Mouse	Goat	Alexa Fluor 594	ThermoFisher Scientific/A11005

Protocol for *in situ* hybridisation in sections

in situ hybridization (ISH) was performed in sections following an adapted protocol (Lauter et al., 2011). The probes were diluted in hybridization buffer (50% deionized formamide, 5xSSC, 5 mg/mL RNA from yeast (Roche), 50 μg/mL heparin sodium salt, 0.1% Tween-20, 5% (v/v) dextran sulfate) and added to the slide with the thawed cryosections, covered with a coverslip and incubated in a humidified chamber at 60°C for 14-16 hours. After hybridization, the slides were washed 2 x 30 min in 50% Formamide/2X SSC/0.1% Tween-20 (SSCT), 15 min in 2X SCCT and 2 x 30 min in 0.2X SCCT, always at 60°C. In sections that were developed using a colorimetric reaction (Fluo-labelled *patched2* and DIG-labelled *foxj1a* probe) the slides were washed 3 x 5 min in PBS/0.1% Tween-20 ($PBST_{20}$) and blocked in 2mg/ml BSA / 2% sheep serum / $PBST_{20}$ for 1 hour at RT. The slides were then incubated with anti-Fluo-AP antibody (1:4000; Roche, 11426338910) at 4°C overnight, followed by 6 x 20 min washes with $PBST_{20}$ and 3 x 5 min washes with 100mM Tris-HCl pH9.5 / 50mM $MgCl_2$ / 100mM NaCl / 0.1% Tween-20. The sections were incubated in BM Purple AP substrate (Roche, 11442074001) or NBT/BCIP (Roche, 11383213001/11383221001) at RT in the dark until signal was developed, fixed 20 min in 4% PFA and mounted. In sections developed with a fluorescent reaction (FISH) the slides were washed 3 x 5 min in TNT (0.1M Tris pH7.5 / 0.15M NaCl / 0.1% Tween-20) and blocked 1 hour at RT in 8% sheep serum/TNT, followed by the incubation with anti-DIG-POD antibody diluted in blocking solution (1:500; Roche, 11207733910) at 4°C overnight. After 6 x 20 min washes with TNT, the hybridized probes were developed with the TSA Plus TMR/Fluorescein system (PerkinElmer, NEL756001KT) following the manufacturer’s protocol. To counterstain the nuclei the sections were incubated with 1µg/ml DAPI in $PBST_{20}$ and mounted in Mowiol medium. In some cases, the FISH was followed by immunostaining against GFP or PCNA. In these sections, after the development with the TSA plus system, the slides were blocked in 1% BSA / PBSTx for 1 hour (preceded by antigen retrieval in the case of the PCNA antibody), followed by incubation with the primary antibody overnight at 4°C. After incubation with the secondary antibody for 2 hours at RT and counterstaining with DAPI, the slides were mounted in Mowiol.
